# A critical review of the epidemiology of Agent Orange/TCDD and prostate cancer

**DOI:** 10.1007/s10654-014-9931-2

**Published:** 2014-07-27

**Authors:** Ellen T. Chang, Paolo Boffetta, Hans-Olov Adami, Philip Cole, Jack S. Mandel

**Affiliations:** 1 Health Sciences Practice, Exponent, Inc., 149 Commonwealth Drive, Menlo Park, CA 94025 USA; 2 Division of Epidemiology, Department of Health Research and Policy, Stanford University School of Medicine, Stanford, CA USA; 3 Institute for Translational Epidemiology and the Tisch Cancer Institute, Icahn School of Medicine at Mount Sinai, New York, NY USA; 4 Department of Epidemiology, Harvard School of Public Health, Boston, MA USA; 5 Department of Epidemiology, University of Alabama at Birmingham, Birmingham, AL USA

**Keywords:** Prostate cancer, TCDD, Dioxin, Agent Orange, Veterans, Epidemiology

## Abstract

To inform risk assessment and regulatory decision-making, the relationship between 2,3,7,8-tetrachlorodibenzo-*p*-dioxin (TCDD) and prostate cancer requires clarification. This article systematically and critically reviews the epidemiologic evidence on the association between exposure to TCDD or Agent Orange, a TCDD-contaminated herbicide used during the Vietnam War, and prostate cancer risk. Articles evaluated include 11 studies of three cohorts, four case–control or cross-sectional studies, and three case-only studies of military veterans with information on estimated Agent Orange or TCDD exposure; 13 studies of seven cohorts, one case–control study, and eight proportionate morbidity or mortality studies of Vietnam veterans without information on Agent Orange exposure; 11 cohort studies of workers with occupational exposure to TCDD; and two studies of one community cohort with environmental exposure to TCDD. The most informative studies, including those of Vietnam veterans involved in Agent Orange spraying or other handling, herbicide manufacturing or spraying workers with occupational TCDD exposure, and community members exposed to TCDD through an industrial accident, consistently reported no significant increase in prostate cancer incidence or mortality. Only some potentially confounded studies of Vietnam veterans compared with the general population, studies with unreliable estimates of Agent Orange exposure, and analyses of selected subgroups of Vietnam veterans reported positive associations. Overall, epidemiologic research offers no consistent or convincing evidence of a causal relationship between exposure to Agent Orange or TCDD and prostate cancer. More accurate exposure assessment is needed in large epidemiologic studies to rule out a causal association more conclusively.

**Electronic supplementary material:**

The online version of this article (doi:10.1007/s10654-014-9931-2) contains supplementary material, which is available to authorized users.

## Introduction

Prostate cancer is the most common non-skin malignancy in U.S. men and the second most common cancer in men globally [1]. Despite extensive clinical and epidemiological research, the causes of prostate cancer remain elusive. Several environmental agents have been proposed to contribute to prostate carcinogenesis, including nutritional factors such as lycopene [2], tobacco smoking [3], and synthetic endocrine-disrupting chemicals [4]. In particular, exposure to 2,3,7,8-tetrachlorodibenzo-*p*-dioxin (TCDD) has been proposed as a possible cause of prostate cancer [5]. In 1997 and 2012, the International Agency for Research on Cancer (IARC) classified TCDD as an established human carcinogen (IARC group 1) [6, 7]. According to the IARC evaluation, the strongest evidence of an increased risk of cancer in humans is for all cancers combined, while “a positive association has been observed” for soft-tissue sarcoma, non-Hodgkin lymphoma, and lung cancer [7]. The classification of TCDD as a group 1 carcinogen is based in large part on mechanistic data showing tumor promotion through modification of cell replication and apoptosis mediated through the aryl hydrocarbon (Ah) receptor and related signaling pathways and responses, which are conserved across species [6, 7].

Results regarding the association between TCDD exposure and risk of prostate cancer were presented from only three epidemiologic studies reviewed in the earlier IARC evaluation in 1997 [6]; none of these detected a significant increase in prostate cancer incidence or mortality among workers potentially exposed to TCDD [8–10]. The 2012 IARC review, which addressed new information published since the 1997 review as part of a broad update on all group 1 carcinogens, did not mention prostate cancer as an endpoint considered in relation to TCDD [7]. Other national and international agencies that have reviewed the epidemiologic evidence on TCDD and cancer, including some that classified TCDD as “carcinogenic to humans” [11], “at least ‘likely to be carcinogenic to humans’” [12], or “known to be a human carcinogen” [13], have likewise largely been silent on prostate cancer [11, 12, 14–16]. Only one of these reports [15] cited any results for prostate cancer, noting that this outcome was not increased above expectation in a pooled international cohort of workers exposed to phenoxy herbicides and chlorophenols contaminated with TCDD [17]. Thus, although epidemiologic studies of the association between TCDD and risk of all cancers and certain site-specific cancers have been critically reviewed, albeit with conflicting conclusions [18–22], the relationship of TCDD with prostate cancer risk has not yet been well characterized.

Since 1991, the U.S. Institute of Medicine (IOM) has been congressionally mandated to conduct a comprehensive evaluation, updated biennially, of scientific and medical information related to health outcomes associated with Agent Orange and other herbicides used for defoliation and crop destruction during the Vietnam War between 1962 and 1971 [23]. Agent Orange is a defoliant made from equal parts of the phenoxy herbicides 2,4-dichlorophenoxyacetic acid (2,4-D) and 2,4,5-trichlorophenoxyacetic acid (2,4,5-T), the latter of which is contaminated with TCDD as a byproduct of the manufacturing process. Since its initial report in 1994, the IOM committee has repeatedly concluded that there is “limited/suggestive evidence of an association” between herbicides and prostate cancer—that is, that “[e]vidence is suggestive of an association between herbicides and the outcome but is limited because chance, bias, and confounding could not be ruled out with confidence” [23–32].

Understanding the association between TCDD and prostate cancer is important for risk assessment and regulatory decision-making, and is also of public health relevance given that low-dose exposure to TCDD is ubiquitous and prostate cancer is one of the most common malignancies worldwide. Furthermore, recent articles focusing on the relationship between Agent Orange exposure and prostate cancer risk have attracted attention to this issue [33, 34]. Therefore, this article aims to provide a detailed review of the epidemiologic evidence on Agent Orange/TCDD and prostate cancer.

## Epidemiology of prostate cancer

More than 1,100,000 new cases of prostate cancer and 300,000 prostate cancer deaths occur each year worldwide [1]. The disease primarily affects older men, with a median age at diagnosis around age 70 years. Incidence rates vary by more than 30-fold internationally, with the highest rates found in Australia and New Zealand, northern and western Europe, and North America, and the lowest rates found in southern and eastern Asia and North Africa [1]. Much of the excess incidence in economically developed regions, along with the rapid rise in prostate cancer incidence in the early 1990s, is attributed to the widespread practice of prostate-specific antigen (PSA) testing to detect asymptomatic tumors, many of which are clinically indolent and would not otherwise become clinically apparent [35, 36]. The high proportion of such latent disease makes prostate cancer particularly difficult to study epidemiologically. That is, any factor that affects diagnostic intensity and particularly PSA testing will inevitably affect observed prostate cancer incidence, making it difficult to distinguish between factors that influence disease development and those that influence disease detection.

The general epidemiology of prostate cancer has been reviewed in other publications [37–41]. The only established risk factors for prostate cancer are older age, family history of the disease (which confers a twofold to fourfold increased risk in first-degree relatives), certain genetic variants, and race, with higher risk among men of black/sub-Saharan African origin than men of white/European origin, and lowest risk among those of Asian origin. Other reviews cited in this section are focused on specific potential risk factors for prostate cancer. A few rare genetic mutations, including variants in the tumor suppressor gene *BRCA2*, the DNA repair genes *PALB2*, *BRIP1*, *CHEK2*, and *NBS1*, and the transcription factor gene *HOXB13*, appear to confer a "moderate" excess risk of prostate cancer, and 76 common variants that confer a "small" excess risk have been identified by genome-wide association studies thus far, together explaining approximately 30 % of the familial risk of prostate cancer [42].

Diet has been extensively studied with respect to prostate cancer risk, with largely inconsistent findings other than inverse associations with lycopene and selenium and a positive association with calcium—although even these associations are not generally accepted as causal [2]. If heavy alcohol consumption or tobacco smoking increases prostate cancer risk—a question that is not settled—the excess risk is probably below 30 % [3, 43]. The role of energy balance, including physical activity, adiposity, and levels of insulin and insulin-like growth factors, in prostate cancer development has also been widely studied, with suggestive but ultimately inconclusive results [2, 44]. A farming occupation appears to be associated with increased prostate cancer risk [45], although results are heterogeneous across studies, and the underlying explanations for this association are unclear given inconsistent associations with specific pesticides [46]. Higher circulating androgen levels may be associated with increased prostate cancer risk, but this apparently complex relationship is not yet understood, and circulating testosterone levels measured in epidemiologic studies may not reflect locally bioavailable levels [47]. Infections have been inconsistently associated with prostate cancer risk [48], including variable findings for sexually transmitted infections, depending on the particular agent and method of detection [49]. Associations with additional causes and biomarkers of inflammation also imply an etiologic role of chronic intraprostatic inflammation in prostate carcinogenesis [50]. No clear association has been demonstrated between ionizing radiation [51], vasectomy [52], or sexual behavior [53] and prostate cancer risk. Uncontrolled confounding by these unestablished risk factors is not likely to be a major source of bias. Instead, potential differences in diagnostic intensity are of greater concern in most epidemiologic studies of prostate cancer risk [54–56].

## Methods

Studies included in this review were selected in a manner similar to that previously described by Boffetta et al. [21] in a review on TCDD and cancer. Specifically, this review focuses on two sets of studies. The first set comprises studies that evaluated prostate cancer incidence or mortality among Vietnam veterans who were involved in the spraying or other handling of Agent Orange, or who reported having been exposed to Agent Orange. Studies of Vietnam veterans without information on Agent Orange/TCDD exposure are also discussed briefly to provide context for the interpretation of studies with such exposure information. The second set comprises studies of prostate cancer incidence or mortality among workers involved in the production or use of herbicides potentially contaminated by TCDD. We also evaluated studies of groups exposed via industrial accidents to herbicides or intermediates potentially contaminated with TCDD.

We did not review studies of workers exposed to herbicides not contaminated by TCDD, agents contaminated by polychlorinated dibenzo-*para*-dioxins other than TCDD, or unspecified or poorly specified combinations of pesticides or herbicides. We also excluded studies of occupational groups with potential but unmeasured exposure to TCDD, such as farmers, forestry workers, pulp and paper workers, chloralkali workers, waste incinerator workers, steel mill workers, and fishermen, because no epidemiologic studies of these groups have aimed specifically at estimating the health effects of TCDD as distinct from other potentially hazardous occupational exposures. Furthermore, average TCDD exposure levels in these groups of workers have been shown or are expected to be relatively low [6]. We also excluded ecologic studies that lack individual-level assessment of exposures and outcomes. Studies of PSA levels in the absence of malignancy (e.g., [57]) or after prostate cancer diagnosis (e.g., [58]) were not considered.

Relevant studies were identified from the IARC Monographs [6, 7], the IOM reports [23–32], and searches of the PubMed database using keywords such as “dioxin,” “TCDD,” “Agent Orange,” “Vietnam,” “herbicides,” “cancer,” “malignancy,” “tumor,” “adenocarcinoma,” “mortality,” “morbidity,” and “prostate.” We also identified additional studies from reference lists of included articles.

## Review of epidemiologic studies

The results of epidemiologic studies of veterans with estimated exposure to Agent Orange/TCDD are provided in Table 1. The results of epidemiologic studies of Vietnam veterans without quantitative or qualitative estimates of Agent Orange/TCDD exposure are provided in Table 2. The results of epidemiologic studies of manufacturers and sprayers of herbicides potentially contaminated with TCDD are provided in Table 3, and the results of epidemiologic studies of the Seveso, Italy, industrial accident involving TCDD are provided in Table 4. All relevant results are provided in the tables; for readability, only selected illustrative results are provided in the text below.

**Table 1:** Epidemiologic studies of veterans with estimated Agent Orange/TCDD exposure

Author	Study subjects	Period of exposure and follow-up	Definition of outcome	Definition of exposure	Level of exposure (with N cases)	RR (95 % CI)	Comments
*Air Force Health Study*							
Wolfe et al. [66]	995 male U.S. Air Force veterans of Operation Ranch Hand (mean age at follow-up = 48.6 years) and 1,299 randomly selected comparison male U.S. Air Force veterans involved in other C-130 aircraft missions in Southeast Asia (mean age at follow-up = 48.4 years), matched on date of birth, race, rank, and military occupation	Active service between August 1961 and May 1972; spray operations between January 1962 and October 1971 Morbidity follow-up between 1982 and 1987 physical examinations	Prostate cancer diagnosed by a doctor, as reported on an in-person questionnaire conducted by interviewers blinded to exposure status; diagnoses verified by medical record retrieval and review; also conducted physical examinations in 1982, 1985, and 1987, but no “invasive procedures to detect evidence of systemic cancer”	Service in Operation Ranch Hand *Median serum TCDD levels* Ranch Hands (N = 888): 12.4 ppt Flying officers (pilot): 7.3 ppt Flying officers (navigator): 9.3 ppt Nonflying officers: 6.6 ppt Flying enlisted personnel: 17.2 ppt Nonflying enlisted personnel: 23.6 ppt Comparison subjects (N = 856): 4.2 ppt Flying officers (pilot): 4.7 ppt Flying officers (navigator): 4.5 ppt Nonflying officers: 4.3 ppt Flying enlisted personnel: 4.0 ppt Nonflying enlisted personnel: 3.9 ppt	Ranch Hands: 2 cases Comparison subjects: 5 cases	NR	*Participation in 1987 examination and questionnaire* 84 % of eligible Ranch Hands; 90 % of Ranch Hands who participated at baseline 75 % of eligible comparison subjects; 91 % of comparison subjects who participated at baseline
Michalek et al. (67)	1,261 male U.S. Air Force veterans of Operation Ranch Hand and 19,101 comparison male U.S. Air Force veterans involved in other C-130 aircraft missions in Southeast Asia	Mortality follow-up from first qualifying tour of duty through December 31, 1987 See Wolfe et al. [66] for exposure period	Genitourinary cancer mortality ascertained using U.S. Air Force, VA Beneficiary Identification Records Locator Subsystem, and Internal Revenue Service records; underlying cause of death on death certificate (obtained for 99.8 % of Ranch Hands and 99.9 % of comparison subjects) classified according to National Center for Health Statistics decision tables	Service in Operation Ranch Hand See Wolfe et al. [66] for median serum TCDD levels	Ranch Hands: 1 death (4.0 per 100,000 person-years) Comparison subjects: 8 deaths (2.1 per 100,000 person-years)	NR	–
Ketchum and Akhtar [62]	See Michalek et al. (67)	Mortality follow-up from first qualifying tour of duty through December 31, 1993 See Wolfe et al. [66] for exposure period	Prostate cancer mortality See Michalek et al. (67) for methods	Service in Operation Ranch Hand *Median serum TCDD levels* Ranch Hands (N = 968 alive, 23 dead) Alive: 26.7 ppt Dead: 35.0 ppt (nonsignificant difference)	Ranch Hands: 2 deaths Expected based on comparison subjects: 0.629 deaths	NR SMR = 3.2 (0.4–11.5)	TCDD assays administered to 1,008 (80 %) of 1,261 Ranch Hands; 991 with quantifiable results Expected deaths calculated with adjustment for date of birth, 5-year age group, rank (officer or enlisted), and military occupation (flyer or nonflyer)
Ketchum et al. [64]	980 male U.S. Air Force veterans of Operation Ranch Hand (mean age at most recent physical examination = 54.6 years for background TCDD, 54.6 years for low TCDD, 50.9 years for high TCDD) and 1,275 randomly selected comparison male U.S. Air Force veterans involved in other C-130 aircraft missions in Southeast Asia (mean age at most recent physical examination = 53.5 years), matched on date of birth, race, rank, and military occupation	Cancer incidence and mortality follow-up from end of service in Southeast Asia through July 10, 1997 See Wolfe et al. [66] for exposure period	Prostate cancer ascertained from periodic physical examinations and questionnaires in 1982, 1985, 1987, and 1992, telephone interviews between 1992 and 1997, medical records (to validate reported diagnoses and identify unreported diagnoses), and death certificates; examinations conducted and records reviewed by personnel blinded to exposure status	TCDD levels measured in serum in 1987 (most) or 1992; levels extrapolated to end of service in Southeast Asia assuming constant half-life of 8.7 years; categorized as “comparison” or “background” (≤10 ppt), “low” (10–≤94 ppt), or “high” (>94 ppt)	Ranch Hands, background TCDD (N = 421): 7 cases Ranch Hands, low TCDD (N = 276): 8 cases Ranch Hands, high TCDD (N = 283): 4 cases Comparison subjects (N = 1,275): 33 cases	Background TCDD: OR = 0.4 (0.1–0.8) Low TCDD: OR = 0.6 (0.3–1.5) High TCDD: OR = 0.7 (0.2–2.2) *P* for trend = 0.41	1,109 Ranch Hands compliant at any examination 100 missing TCDD 17 with nonquantifiable TCDD 1 reported cancer not verified by medical record 11 with cancer before service in Southeast Asia 1,493 comparison subjects compliant at any examination 140 missing TCDD 50 with nonquantifiable TCDD 25 with dioxin >10 ppt 3 with cancer before service in Southeast Asia ORs adjusted for birth year, military occupation, race, percent body fat at time of dioxin blod draw, lifetime cigarette smoking, lifetime alcohol consumption, and exposure to asbestos, ionizing radiation, industrial chemicals, herbicides, insecticides, and degreasing chemicals
Kayajanian et al. [65]	See Ketchum et al. [64]	See Ketchum et al. [64]	See Ketchum et al. [64]	TCDD body burden modeled as partially described in Kayajanian [64], categorized into seven groups with break points chosen to identify “significant cancer incidence peaks and valleys”: 0 ppt, 1.25–2.50 ppt, 2.51–4.00 ppt, 4.01–8.00 ppt, 8.01–10.00 ppt, 27.18–127.45 ppt, and 128.13–3,290.18 ppt All U.S. Air Force veterans in Southeast Asia (including Operation Ranch Hand and non-Ranch Hand veterans) categorized by serum TCDD and compared with U.S. national cancer incidence rates	0 ppt (N = 117 whites, 9 blacks): 1 white case, 3 black cases 1.25–2.50 ppt (N = 150 whites, 14 blacks): 1 white case, 1 black case 2.51–4.00 ppt (N = 452 whites, 22 blacks): 9 white cases, 2 black cases 4.01–8.00 ppt (N = 749 whites, 40 blacks): 20 white cases, 0 black cases 8.01–10.00 ppt (N = 134 whites, 9 blacks): 4 white cases, 0 black cases 27–127 ppt (N = 308 whites, 27 blacks): 8 white cases, 1 black case ≥128 ppt (N = 214 whites, 10 blacks): 2 white cases, 0 black cases	0 ppt Whites: SIR = 2.9; Blacks: SIR = 133.3 1.25–2.50 ppt Whites: SIR = 3.1; Blacks: SIR = 54.5 2.51–4.00 ppt Whites: SIR = 7.4; Blacks: SIR = 33.8 4.01–8.00 ppt Whites: SIR = 6.4; Blacks: SIR = 0 8.01–10.00 ppt Whites: SIR = 6.1; Blacks: SIR = 0 27–127 ppt Whites: SIR = 7.2; Blacks: SIR = 10.5 ≥128 ppt Whites: SIR = 4.2; Blacks: SIR = 0 1.25–4.00 ppt Blacks: SIR = 38.7, *P* < 0.10 versus 0 ppt ≥4.01 ppt Blacks: SIR = 3.6, *P* < 0.06 versus 1.25–4.00 ppt, *P* < 0.002 versus 0 ppt	–
Akhtar et al. [63]	1,189 male U.S. Air Force veterans of Operation Ranch Hand (median age in 1982 = 45.1 years for background TCDD, 45.5 years for low TCDD, 37.2 years for high TCDD; N = 1,009 for internal comparisons) and 1,776 randomly selected comparison male U.S. Air Force veterans involved in other C-130 aircraft missions in Southeast Asia (median age in 1982 = 43.3 years; N = 1,429 for internal comparisons), matched on date of birth, race, rank, and military occupation External comparisons with U.S. male national cancer incidence rates (from National Cancer Institute) and mortality rates (from National Center for Health Statistics) standardized by age, race, and calendar year	Cancer incidence and mortality follow-up from end of service in Southeast Asia through December 31, 1999 See Wolfe et al. [66] for exposure period	Prostate cancer ascertained from periodic questionnaires and physical examinations (including rectal examination in 1982, 1985, 1987, 1992, and 1997, and PSA in 1992 and 1997), medical records, and death certificates; examinations conducted and records reviewed by medical and support staff blinded to exposure status	External comparisons: U.S. Air Force service in Southeast Asia between 1961 and 1972 Internal comparisons: TCDD levels measured on a lipid weight basis in serum in 1987, 1992, or 1997; levels extrapolated to end of service in Vietnam assuming constant half-life of 7.6 years; categorized as “background” (≤10 ppt), “low” (>10–≤118.5 ppt), or “high” (>118.5 ppt)	*Ranch Hands* 36 cases in whites 24.71 cases expected based on U.S. rates 2 deaths in whites 2.84 deaths expected based on U.S. rates *Comparison subjects* 54 cases in whites 33.34 cases expected based on U.S. rates 3 deaths in whites 3.91 deaths expected based on U.S. rates	*Ranch Hands* Whites: SIR = 1.46 (1.04–2.00) Whites, tours ending 1966–1970: SIR = 1.68 (1.19–2.33) Whites, ≤2 years in Southeast Asia: SIR = 1.54 (0.98–2.32) Whites, 100 % of Southeast Asia service in Vietnam: SIR = 1.66 (1.00–2.60) SMR = 0.69 (0.12–2.33) *Comparison subjects* Whites: SIR = 1.62 (1.23–2.10), *P* = 0.62 versus Ranch Hands Whites, tours ending 1966–1970: SIR = 1.64 (1.20–2.20), *P* = 0.92 versus Ranch Hands Whites, ≤2 years in Southeast Asia: SIR = 0.68 (0.30–1.35), *P* = 0.05 versus Ranch Hands Whites, 0 % of Southeast Asia service in Vietnam: SIR = 0.59 (0.15–1.61), *P* = 0.08 versus Ranch Hands SMR = 0.77 (0.20–2.09), *P* = 0.93 versus Ranch Hands	*Military tour date categories* Before 1962 or after 1972: no herbicide 1962–1965: pre-Agent Orange 1966–1970: predominantly Agent Orange 1971–1972: post-Agent Orange 53 of 90 prostate cancer cases (25 Ranch Hands, 28 comparison subjects) were diagnosed as a direct result of study-specific physical examinations SIRs standardized by 5-year age and 5-year calendar period RRs for TCDD and prostate cancer in whites with <2 years of service in Southeast Asia adjusted for age at tour, military occupation, pack-years of cigarette smoking, and logarithm of years served in Southeast Asia (if appropriate)
Akhtar et al. [63]	See above	See above	See above	See above	*Restricted to whites with ≤2 years in Southeast Asia* Ranch Hands, background TCDD (N = 287): 10 cases Ranch Hands, low TCDD (N = 151): 6 cases Ranch Hands, high TCDD (N = 174): 5 cases Comparison subjects (N = 580): 7 cases	*Whites with ≤2 years in Southeast Asia* Background TCDD: RR = 1.50 (0.51–4.40) Low TCDD: RR = 2.17 (0.68–6.87) High TCDD: RR = 6.04 (1.48–24.61) RR per 1-unit increase in log TCDD: RR = 1.48 (0.93–2.35)	1,196 Ranch Hands compliant at any examination 7 with cancer before service in Southeast Asia 34 dead before first dioxin blood draw 120 noncompliant after 1985 10 medically deferred from blood draw 8 refused blood draw 1 unable to draw blood 2 without blood for unknown reason 4 failed assay quality control check(s) 1 with missing lipids 1,785 comparison subjects compliant at any examination 6 with cancer before service in Southeast Asia 3 with cancer during service in Southeast Asia 39 dead before first dioxin blood draw 268 noncompliant after 1985 2 not locatable after 1985 15 medically deferred from blood draw 13 refused blood draw 10 failed assay quality control check(s)
Akhtar et al. [63]	See above	See above	See above	See above	*Restricted to whites, Ranch Hands with 100 % of Southeast Asia service in Vietnam and comparison subjects with 0 % of Southeast Asia service in Vietnam* Ranch Hands, background TCDD (N = 252): 9 cases Ranch Hands, low TCDD (N = 132): 4 cases Ranch Hands, high TCDD (N = 165): 4 cases Comparison subjects (N = 291): 3 cases	*Whites, Ranch Hands 100% in Vietnam and comparison subjects 0% in Vietnam* Background TCDD: RR = 2.48 (0.38–16.07) Low TCDD: RR = 2.36 (0.35–16.01) High TCDD: RR = 4.67 (0.75–29.07) RR per 1-unit increase in log TCDD: RR = 1.07 (0.64–1.78)	See above
Pavuk et al. [72]	1,482 comparison male U.S. Air Force veterans (median age at qualifying tour = 28.8 years) involved in C-130 aircraft missions other than Operation Ranch Hand, primarily transport missions, in Southeast Asia between 1961 and 1971 (i.e., comparison subjects); stationed mostly in Taiwan, Philippines, Guam, Japan, and Thailand, with an average of 23 % of Southeast Asia service in Vietnam	Active service between 1961 and 1971 (overall Southeast Asia tours of duty between 1942 and 1982) Cancer incidence and mortality follow-up from January 1, 1982, through December 31, 2003	Prostate cancer ascertained from periodic physical examinations and questionnaires in 1982, 1985, 1987, 1992, 1997, and 2002, medical records, and death certificates; examinations conducted and records reviewed by personnel blinded to exposure status	TCDD levels measured on a lipid weight basis in serum in 1987 (most), 1992, or 1997; categorized into quartiles	TCDD quartile 1 (0.4–2.6 pg/g lipid; N = 370): 13 cases TCDD quartile 2 (2.6–3.8 pg/g lipid; N = 373): 24 cases TCDD quartile 3 (3.8–5.2 pg/g lipid; N = 369): 24 cases TCDD quartile 4 (5.2–54.8 pg/g lipid; N = 370): 22 cases	TCDD quartile 2 versus 1: RR = 1.7 (0.8–3.3) TCDD quartile 3 versus 1: RR = 1.5 (0.7–2.9) TCDD quartile 4 versus 1: RR = 1.2 (0.6–2.4) RR per 1-pg/g lipid increase in TCDD: RR = 1.1 (0.7–1.5)	1,853 comparison subjects 212 not compliant with any physical examination 119 with missing serum TCDD 40 with cancer before 1982 RRs for TCDD and prostate cancer adjusted for military occupation, race, year of birth, number of years served in Southeast Asia, body mass index at the qualifying tour, relative change in body mass index from qualifying tour to TCDD measurement, and baseline pack-years of cigarette smoking history RRs for years served and prostate cancer adjusted for military occupation, race, year of birth, body mass index at the qualifying tour, and baseline pack-years of cigarette smoking history; no change after additional adjustment for serum TCDD No interaction between serum TCDD and years served in Southeast Asia (*P* = 0.62)
Pavuk et al. [72]	See above	See above	See above	Years served in Southeast Asia ascertained from military records; categorized into quartiles	Years in Southeast Asia quartile 1 (0.1–1.3 years; N = 367): 8 cases Years in Southeast Asia quartile 2 (1.3–2.1 years; N = 373): 11 cases Years in Southeast Asia quartile 3 (2.1–3.7 years; N = 371): 18 cases Years in Southeast Asia quartile 4 (3.7–16.4 years; N = 370): 36 cases	Years in Southeast Asia quartile 2 versus 1: RR = 1.3 (0.5–3.2) Years in Southeast Asia quartile 3 versus 1: RR = 2.2 (1.0–4.9) Years in Southeast Asia quartile 4 versus 1: RR = 2.4 (1.1–5.2) RR per 1-year increase in years in Southeast Asia: RR = 1.1 (1.0–1.2)	See above
Pavuk et al. [73]	1,019 male U.S. Air Force veterans of Operation Ranch Hand (mean age at qualifying tour = 29.8 years) and 1,497 randomly selected comparison male U.S. Air Force veterans involved in other C-130 aircraft missions in Southeast Asia (mean age at qualifying tour = 29.9 years), matched on date of birth, race, rank, and military occupation	Cancer incidence follow-up from January 1, 1982, through December 31, 2003; average follow-up = 21 years; no loss to follow-up See Wolfe et al. [66] for exposure period	Prostate cancer coded from medical records; some reports based on physical examinations and questionnaires in 1982, 1985, 1987, 1992, 1997, and 2002	TCDD levels measured in serum in 1987 (most) and 1992, 1997, and 2003; estimated half-life (calculated from 343 veterans with an average of 4 repeated TCDD measurements) used to calculate total cumulative TCDD level as area under the curve (AUC) from end of exposure in Vietnam to end of follow-up If serum TCDD was <10 ppt in 1987, AUC was calculated by multiplying measured TCDD levels by years of follow-up Median 20-year cumulative TCDD (443 ppt-years) used to categorize Ranch Hands into lower and higher exposure categories Median TCDD in 1987 = 11.6 ppt for Ranch Hands, 3.86 ppt for comparison subjects Median 20-year cumulative TCDD = 433 ppt-years for Ranch Hands, 77.2 ppt-years for comparison subjects	Comparison subjects (N = 1,497): 81 cases 17 cases in Southeast Asia before 1969 64 cases in Southeast Asia after 1969 16 cases served <2 years 65 cases served ≥2 years Lower TCDD (N = 509): 31 cases 9 cases in Southeast Asia before 1969 22 cases in Southeast Asia after 1969 20 cases served <2 years 11 cases served ≥2 years Higher TCDD (N = 510): 28 cases 15 cases in Southeast Asia before 1969 13 cases in Southeast Asia after 1969 14 cases served <2 years 14 cases served ≥2 years	*Overall* Lower TCDD: RR = 1.02 (0.67–1.55) Higher TCDD: RR = 1.22 (0.79–1.89) *P* for trend = 0.42 *In Southeast Asia before 1969* Lower TCDD: RR = 1.00 (0.44–2.27) Higher TCDD: RR = 2.27 (1.11–4.66) *P* for trend = 0.04 *In Southeast Asia after 1969* Lower TCDD: RR = 1.09 (0.67–1.79) Higher TCDD: RR = 0.85 (0.46–1.55) *P* for trend = 0.75 *Served <2* *years in Southeast Asia* Lower TCDD: RR = 1.87 (0.96–3.65) Higher TCDD: RR = 2.15 (1.03–4.48) *P* for trend = 0.03 *Served ≥2* *years in Southeast Asia* Lower TCDD: RR = 0.76 (0.40–1.45) Higher TCDD: RR = 1.05 (0.59–1.90) *P* for trend = 0.89	1,197 Ranch Hands (62 cases) compliant at any examination 170 (1 case) without serum TCDD 6 (1 case) with <20 years of follow-up 1 (1 case) with death or prostate cancer before 1982 1 (0 cases) with missing covariate(s) 1,854 comparison subjects (89 cases) compliant at any examination 331 (3 cases) without serum TCDD 14 (4 cases) with <20 years of follow-up 3 (1 case) with death or prostate cancer before 1982 9 (0 cases) with missing covariate(s) RRs adjusted for age at qualifying tour, body mass index at qualifying tour, occupation, and pack-years of cigarette smoking at baseline; no change after additional adjustment for age
Pavuk et al. [73]	See above	See above	See above	See above	*Comparison subjects* 35 cases ≤77.2 ppt-years TCDD 46 cases >77.2 ppt-years TCDD 18 cases ≤789 days in Southeast Asia 63 cases >789 days in Southeast Asia 17 cases <1969 duty in Southeast Asia 64 cases ≥1969 duty in Southeast Asia *Ranch Hands* 31 cases ≤434 ppt-years TCDD 28 cases >434 ppt-years TCDD 24 cases ≤426 days in Southeast Asia 35 cases >426 days in Southeast Asia 24 cases <1969 duty in Southeast Asia 35 cases ≥1969 duty in Southeast Asia	*Comparison subjects* >77.2 versus ≤77.2 ppt-years TCDD: RR = 1.10 (0.69–1.75) >789 versus ≤789 days in Southeast Asia: RR = 2.18 (1.27–3.76) ≥1969 versus <1969 duty in Southeast Asia: RR = 1.47 (0.86–2.52) *Ranch Hands* >434 versus ≤434 ppt-years TCDD: RR = 1.32 (0.75–2.34) >426 versus ≤426 days in Southeast Asia: RR = 0.97 (0.56–1.67) ≥1969 versus <1969 duty in Southeast Asia: RR = 0.88 (0.52–1.48)	Gleason scores in Ranch Hand cases: 4 (6.8 %) missing; 5 (8.5 %) 0–4; 34 (57 %) 5–6; 16 (27 %) ≥7 Gleason scores in comparison cases: 11 (13.4 %) missing; 6 (7.3 %) 0–4; 37 (46 %) 5–6; 27 (33 %) ≥7; *P* = 0.44 Gupta et al. [74] reported an inverse association between serum TCDD and risk of benign prostatic hyperplasia in comparison subjects, but not Ranch Hand veterans, and an inverse association between serum TCDD and serum testosterone in both groups
*Army Chemical Corps*							
Thomas and Kang [83]	894 male veterans who served in at least one U.S. Army Chemical Corps unit assigned to Vietnam between 1966 and 1971 (mean age at end of service in Vietnam = 25 years), identified from morning reports by the U.S. Army and Joint Services Environmental Support Group Comparisons with U.S. male national mortality rates standardized by age, race, and calendar year No comparisons for morbidity data	Assigned to Vietnam between 1966 and 1971 Mortality follow-up from date last served in Vietnam through December 31, 1987; mean follow-up = 18 years	Prostate cancer mortality ascertained from VA Beneficiary Identification Records Locator Subsystem, Social Security Administration, U.S. Internal Revenue Service, National Death Index, and military records; underlying cause of death coded from death certificate for all subjects known to be deceased Prostate cancer morbidity ascertained from linkage to the VA Agent Orange Registry of Vietnam veterans who volunteered for a special physical examination at a VA medical center since 1978 (168 matches with study cohort), and Patient Treatment File of inpatient discharges at all 172 U.S. VA medical centers since 1970 (136 matches with study cohort; 257 total matches)	Service in Army Chemical Corps unit assigned to Vietnam between 1966 and 1971	Army Chemical Corps: 0 deaths, 0 cases	NR	954 Army Chemical Corps Vietnam veterans ascertained from morning reports 20 with insufficient identifiers 15 not matched with personnel rosters 16 with missing military records 9 killed in military action In a study of TCDD levels in adipose tissue from 10 Vietnam veterans heavily exposed to herbicides, the two participating Army Chemical Corps veterans had two of the three highest measured TCDD levels (Kahn et al. 75)
Cypel and Kang [87]	2,872 male U.S. Army veterans with ≥1 assignment to the Chemical Corps in Vietnam (mean age at start of follow-up = 24.0 years, end of follow-up = 58.8 years), identified from morning reports, Defense Manpower Data Center, and Army Chemical School rosters; and 2,737 comparison male veterans with last U.S. Army discharge from Chemical Corps, but who never served in Southeast Asia (mean age at start of follow-up = 23.0 years, end of follow-up = 57.1 years) External comparisons with U.S. male national mortality rates standardized by age, race, and calendar year	Active service between July 1, 1965, and March 28, 1973 Mortality follow-up from date of last military service or March 28, 1973 (whichever was earlier), through December 31, 2005; mean follow-up in both cohorts ≈32 years	Prostate cancer mortality ascertained from VA Beneficiary Identification Records Locator Subsystem, Social Security Administration, and National Death Index; underlying cause of death coded from death certificates obtained for decedents through 1991, from National Death Index Plus thereafter Cause of death ascertained for 98 % of Vietnam veterans, 95 % of non-Vietnam veterans	Service in Army Chemical Corps in Vietnam during period of U.S. combat involvement (1965–1973)	Army Chemical Corps in Vietnam: 5 deaths (crude rate = 0.54 per 10,000) Army Chemical Corps outside Southeast Asia: 2 deaths (crude rate = 0.22 per 10,000)	Crude RR = 2.41 Adjusted RR = 1.02 (0.19–5.64) Army Chemical Corps in Vietnam: SMR = 1.05 (0.34–2.45) Army Chemical Corps outside Southeast Asia: SMR = 0.95 (0.12–3.43)	RR adjusted for race, rank, duration of military service, and age at entry into follow-up
*Other studies*							
Zafar and Terris [5]	32 patients with self-reported Agent Orange exposure (mean age = 60.4 years, median = 59 years) and an age-matched control group of 96 patients without self-reported Agent Orange exposure (mean age = 60.6 years, median = 59 years) out of 400 consecutive U.S. veterans referred for prostate needle biopsy during a 30-month period between 1998 and 2000 at a VA Palo Alto Health Care System	Period of exposure NR Prostate biopsy during a 30-month period in 1998–2000	Prostate cancer diagnosed on biopsy after referral	Self-reported Agent Orange exposure as reported before biopsy in a routine questionnaire administered upon medical center registration	*Exposed* 21 cases (40.4 %) 3 with Gleason score ≥7 (14.3 % of 21) 5 cases (29 %) of 17 aged <60 years 1 case (9 %) of 11 aged <50 years *Unexposed* 54 cases (34.6 %) 15 with Gleason score ≥7 (27.8 % of 54) 10 cases (42 %) of 24 aged <60 years 10 cases (33 %) of 30 aged <60 years	% cases: *P* = 0.15; Pearson ρ = 0.06 % cases with Gleason grade ≥7: *P* = 0.41	PSA in exposed: mean = 8.4 ng/mL, median = 6.6 ng/mL, range 1.4–48.7 ng/mL PSA in unexposed: mean = 8.2 ng/mL, median = 5.7 ng/mL, range 0.5–55.2 ng/mL; *t* test *P* = 0.9 Mean length of cancer in biopsy = 3.8 cm in exposed, 4.4 cm in unexposed; *P* = 0.34 At VA facility, an average of 1.07 % of exposed adult male veterans and 1.33 % of unexposed adult male veterans were referred for prostate biopsy annually; 32 exposed referred patients were significantly younger the 400 referred patients overall (mean age = 65.5 years, median age = 66 years)
Giri et al. [92]	47 prostate cancer cases diagnosed at the VA Medical Center in Ann Arbor, Michigan, and born between January 1, 1935, and December 31, 1953 (mean age in 2001 = 55.9 years), and 142 male controls without prostate cancer selected randomly from the General Medicine Clinic, frequency-matched to cases by age, born in same date range (mean age in 2001 = 56.7 years)	Period of exposure NR; birth date restriction designed to encompass “the appropriate age for potential military service in Vietnam” Medical records data collected between June 2000 and July 2001	Prostate cancer identified by a computerized search of pathology records at the medical center	Self-reported Agent Orange exposure, classified based on a review of computerized medical records and each subject’s response to the question, “Exposure to Agent Orange?”; unknown timing of exposure assessment relative to disease diagnosis	*Cases* 11 (23.4 %) exposed 29 (61.7 %) unexposed 7 (14.9 %) unknown (excluded) 14 (29.8 %) with service in Vietnam 23 (48.9 %) white *Controls* 17 (12.0 %) exposed 106 (74.6 %) unexposed 19 (13.4 %) unknown (excluded) 49 (34.5 %) with service in Vietnam 93 (65.5 %) white	OR = 2.06 (0.81–5.23) OR for whites = 2.72 (0.90–8.23) Crude Chi square *P* for Agent Orange = 0.047 Crude Chi square *P* for Vietnam service >0.05	ORs adjusted for age and race Mean age at diagnosis = 55 years in exposed, 56 years in unexposed; *P* > 0.05 Mean Gleason score = 6.5 in exposed, 6.2 in unexposed; *P* > 0.05 Pathologic stage = 5 (62.5 %) organ-confined and 3 (37.5 %) extraprostatic extension (no nodal involvement or metastasis) of 8 exposed, 9 (100 %) organ-confined of 9 exposed; *P* = 0.08
Chamie et al. [34]	All Vietnam-era veterans who received care in the Northern California VA Health System, including 6,214 classified as exposed (mean age = 60.8 years) and 6,930 classified as unexposed (mean age = 61.4 years) to Agent Orange, excluding those stationed outside of the Vietnam theater during period of active duty	Period of exposure = 1962–1971 Prostate cancer follow-up = 1998–2006	Prostate cancer identified through the VA Computerized Patient Record System	Self-reported Agent Orange exposure, based on the initial application for medical benefits and having been “stationed in known areas that were sprayed with Agent Orange during 1962 through 1971” Patients were excluded if they changed self-reported exposure status from unexposed to exposed after prostate cancer diagnosis (N = 7); patients were included if they filed for medical benefits and reported positive exposure status after prostate cancer diagnosis (N = 38)	*239 exposed cases* 74 (32 % of 229 known) Gleason score = 7 52 (23 % of 229 known) Gleason score = 8–10 32 (13.4 %) metastatic on presentation *124 unexposed cases* 47 (38 %) Gleason score = 7 13 (10 %) Gleason score = 8–10 5 (4 %) metastatic on presentation	Crude OR = 2.19 (1.75–2.75) Crude HR = 2.87 (2.31–3.57) Adjusted OR = 4.83 (3.42–6.81) Adjusted OR for high-grade disease = 2.59 (1.30–5.13) Adjusted OR for metastasis at presentation = 4.32 (1.34–13.96) OR (unspecified if crude or adjusted) after exclusion of 38 cases who reported exposure after diagnosis = 1.85 (1.47–2.31)	*Exposed versus unexposed* *veterans* 3.1 versus 3.8 % with finasteride use; *P* = 0.03 71.5 versus 71.7 % with screening PSA; *P* = 0.85 Mean PSA = 3.1 versus 1.8 ng/mL; *P* = 0.11 9.1 versus 6.1 % with PSA > 4 ng/mL; *P* < 0.001 84.3 versus 83.1 % with urologic exam for elevated PSA; *P* = 0.67 81.8 versus 77.7 % with biopsy for elevated PSA; *P* = 0.22 *Exposed versus unexposed cases* Mean age = 59.7 versus 62.2 years; *P* = 0.002 33.9 versus 29.0 % African American; *P* = 0.46 8.8 versus 16.1 % with family history; *P* = 0.05 2.6 versus 3.4 % with finasteride use; *P* = 0.73 Mean PSA = 34.8 versus 19.2 ng/mL; *P* = 0.38 69.9 versus 64.5 % clinical stage T1c; *P* = 0.46 Mean Gleason score = 6.8 versus 6.5; *P* = 0.007 21.8 versus 10.5 % Gleason ≥8; *P* = 0.009 13.4 versus 4 % metastatic; *P* = 0.005 Mean time from Agent Orange exposure to prostate cancer = 407 months (33.9 years) ORs adjusted for preoperative PSA, age at diagnosis, body mass index, race, finasteride use, and smoking history; ORs for high-grade and metastatic disease also adjusted for family history ~500 prostate cancer cases diagnosed before 1998 may have been missed (Schechter et al. 2009)
Ansbaugh et al. [33]	2,720 veterans (203 with Agent Orange exposure, 2,517 without) referred to the Portland VA Medical Center for initial prostate biopsy, without a history of prostate cancer (mean age at biopsy = 64.7 years; cases = 65.7 years, noncases = 64.2 years)	Period of exposure NR Follow-up period NR	Clinical, laboratory, and pathological characteristics recorded on a standardized form, with additional clinical information obtained from the Veterans Integrated Service Network 20 Consumer Health Information Performance Sets Data Warehouse	Agent Orange exposure classified within the VA medical record (from the Veterans Integrated Service Network 20 data warehouse) in accordance with the Portland VA Medical Center standards for documenting exposure, i.e., based on military service in “a location where [Agent Orange] was known to have been used” or self-reported exposure Exposure status determined during patient enrollment into VA hospital system, prior to prostate biopsy; 9 veterans (0.3 %) without available Agent Orange exposure status assumed to be unexposed	74 exposed cases 40 with Gleason score ≥7 822 unexposed cases 419 with Gleason score ≥7 129 exposed noncases 1,695 unexposed noncases	OR for prostate cancer versus none = 1.52 (1.07–2.13) OR for prostate cancer with Gleason score ≥7 versus no or low-grade disease = 1.74 (1.14–2.63) OR for prostate cancer with Gleason score ≥7 versus none = 1.75 (1.12–2.74) OR for prostate cancer with Gleason score <7 versus none = 1.24 (0.81–1.91) OR for prostate cancer with Gleason score ≥8 versus no or low-grade disease = 2.10 (1.22–3.61) [1.22–3.62 in text]	ORs adjusted for age, PSA density, and digital rectal examination results Age at biopsy was significantly younger in exposed than unexposed veterans (60.6 vs. 65.0 years), cases (61.4 vs. 66.1 years), and high-grade cases (62.1 vs. 66.9 years) Mean PSA did not differ significantly between exposed and unexposed veterans (11.2 vs. 12.4 ng/mL), cases (8.8 vs. 23.0 ng/mL), or high-grade cases (7.9 vs. 7.7 ng/mL), excluding values >5,000 ng/mL Mean PSA density did not differ significantly between exposed and unexposed veterans (0.20 vs. 0.19 ng/mL/mL), cases (0.34 vs. 0.34 ng/mL/mL), or high-grade cases (0.30 vs. 0.44 ng/mL/mL), excluding values >20 ng/mL/mL In exposed veterans, military service branch was not significantly associated with prostate cancer risk
Yi et al. [94]	114,562 Korean veterans of the Vietnam War identified by the Ministry of National Defense and Ministry of Government Administration and Home Affairs as of June 2004, who completed a written, mailed survey	Exposure during the Vietnam War Health survey in July 2004	Prevalent prostate cancer, self-reported by mailed questionnaire	Self-reported perceived exposure to Agent Orange in Vietnam based on six yes/no questions on spraying, handling spray equipment, presence during spraying, contact on skin or clothing, walking through sprayed area, or having been exposed in any other way; categorized into high (“yes” to either of first 2 questions), moderate (“yes” to either of second 2 questions), low/none (“yes” to either of last 2 questions or “no”/”don’t know” to all) Exposure opportunity index model E4 developed by Stellman et al. (2003) based on proximity of military unit to the sprayed area; battalion/company-level units identified in self-reported surveys, division/brigade-level units identified from Ministry of Defense military records. Military coordinate information sent to Stellman team for calculating E4 scores, log-transformed for use as Agent Orange exposure index, categorized into low versus high or none, low, moderate, and high In 102 Korean Vietnam veterans with serum TCDD measured in 2007 (mean = 1.2 ppt, median = 0.9 ppt; N = 2 with TCDD > 10 ppt), log-transformed TCDD level was not correlated with division/brigade-level exposure (r^2^ = 0.07), company/battalion-level exposure (r^2^ = 0.16), or self-reported perceived exposure (r^2^ = 0.13) [97]	Self-reported perceived exposure Low: 822 cases High: 1,388 cases Division/brigade-level modeled exposure Low: 858 cases High: 1,049 cases Battalion/company-level exposure Low: 1,080 cases High: 827 cases	*Self-perceived exposure* High versus low: OR = 1.50 (1.37–1.64) Low versus none: OR = 1.26 (1.08–1.45) Moderate versus none: OR = 1.48 (1.32–1.64) High versus none: OR = 1.91 (1.69–2.16) *P* for trend <0.001 *Division/brigade-level modeled exposure* High versus low: OR = 1.01 (0.92–1.11) Low versus none: OR = 1.05 (0.92–1.21) Moderate versus none: OR = 1.08 (0.94–1.23) High versus none: OR = 0.99 (0.86–1.15) *P* for trend = 1.00 *Battalion/company-level modeled exposure* High versus low: OR = 1.05 (0.96–1.15) Low versus none: OR = 1.04 (0.92–1.17) Moderate versus none: OR = 1.04 (0.91–1.19) High versus none: OR = 1.11 (0.97–1.27) *P* for trend = 0.15	187,897 veterans identified 23,689 deceased, emigrated, or with unknown residential status 49,646 (30 %) nonparticipants Self-reported perceived exposure: 40,048 (34.9 %) none 15,093 (13.2 %) low 40,935 (35.7 %) moderate 18,496 (16.1 %) high Division-/brigade-level exposure opportunity index: 19,360 (20.1 %) none 27,091 (28.2 %) low 29.909 (31.1 %) moderate 19,766 (20.6 %) high Battalion/company-level exposure opportunity index: 25,102 (26.1 %) none 31,774 (33.1 %) low 20,684 (21.5 %) moderate 18,566 (19.3 %) high ORs adjusted for age, military rank, smoking, alcohol drinking, physical activity, education, household income, herbicide exposure in Korea, and body mass index
*Case*-*only studies of disease characteristics*							
Everly et al. [100]	81 Vietnam veterans (29 with Agent Orange exposure, 51 without) and 433 consecutive nonveterans of comparable age treated with permanent brachytherapy for prostate cancer between May 1995 and January 2005 at the Schiffler Cancer Center in Wheeling, West Virginia	Exposure during Vietnam War Permanent prostate brachytherapy in 1995–2005	Clinical and pathological characteristics of prostate cancer treated with permanent brachytherapy Biochemical recurrence and survival not shown here; median follow-up = 4.5 years (mean ± SD = 5.0 ± 2.5 years)	Agent Orange exposure as determined by eligibility for VA benefits	*29 (36* *%) exposed cases* mean ± SD: age at implant: 56.6 ± 4.1 years preimplant PSA: 11.7 ± 12.2 ng/mL Gleason score: 6.7 ± 0.9 % positive biopsies: 47.2 ± 30.2 prostate volume: 31.1 ± 8.8 cm^3^ 26 clinical stage T1b–T2c 3 clinical stage ≥T2c 12 with perineural invasion 17 without perineural invasion 9 low-risk 10 intermediate-risk 10 high-risk 2 dead (trauma, lung cancer) *52 (64* *%) unexposed cases* mean ± SD: age at implant: 57.6 ± 3.8 years preimplant PSA: 8.0 ± 9.6 ng/mL Gleason score: 6.6 ± 0.6 % positive biopsies: 36.8 ± 25.4 prostate volume: 21.1 ± 7.0 cm^3^ 50 clinical stage T1b–T2c 2 clinical stage ≥T2c 17 with perineural invasion 35 without perineural invasion 22 low-risk 23 intermediate-risk 7 high-risk 0 dead	NR *P* < 0.05 for age at implant and pretreatment PSA *P* ≥ 0.05 for Gleason score, % positive biopsies, prostate volume, clinical stage, perineural invasion, risk group	“In univariate and multivariate analysis, neither Agent Orange exposure nor veteran status predicted for [cause-specific survival], [biochemical progression-free survival], or [overall survival] (Table 2)”
Li et al. [98]	93 veterans (37 with Agent Orange exposure, 56 without) with prostate cancer treated with radical prostatectomy between April 2005 and September 2009 at the VA Medical Center in Augusta, Georgia	Period of exposure NR Radical prostatectomy in 2005–2009	Clinical and pathological characteristics of prostate cancer treated with radical prostatectomy, with data abstracted from electronic medical records Biochemical recurrence not shown here; median follow-up = 64 months (interquartile range 42–72 months)	Agent Orange exposure as self-reported by patients and/or according to military records confirming service “in an area in which AO had been sprayed” Dioxin-like toxic equivalency values measured in ~ 3 g abdominal subcutaneous adipose tisue obtained intraoperatively during radical prostatectomy; analyzed as a continuous variable or a dichotomous variable with cutoff at median	*37 (40* *%) exposed cases* 8 PSA ≥ 10 ng/mL 8 pathological stage T3 or T4 21 biopsy Gleason score ≥7 19 prostatectomy Gleason score ≥7 10 adverse pathology 5 positive margins 9 extracapsular extension 4 seminal vesicle invasion 3 lymph node metastasis 4 dead *56 (60* *%) unexposed cases* 6 PSA ≥10 ng/mL 6 pathological stage T3 or T4 33 biopsy Gleason score ≥7 34 prostatectomy Gleason score ≥7 23 adverse pathology 20 positive margins 8 extracapsular extension 1 seminal vesicle invasion 0 lymph node metastasis 2 dead	*Exposed versus unexposed* Age: 60 versus 57 years, *P* = 0.01 Black race: 38 versus 65 %, *P* = 0.01 Prostate volume: 48 versus 43 cc, *P* = 0.23 Median PSA: 5.0 versus 5.4 ng/mL, *P* = 0.67 PSA ≥10: 21 versus 10 %, *P* = 0.15 Pathological T3/T4: 22 versus 11 %, *P* = 0.15 Biopsy Gleason score ≥7: 57 versus 59 %, *P* = 0.97 Pathological Gleason score ≥7: 51 versus 61 %, *P* = 0.27 Adverse pathology: 41 versus 41 %, *P* = 0.16 Positive margins: 14 versus 36 %, *P* = 0.02 Extracapsular extension: 24 versus 14 %, *P* = 0.22 Seminal vesicle invasion: 11 versus 2 %, *P* = 0.08 Lymph node metastasis: 8 versus 0 %, *P* = 0.06 Death: 11 versus 4 %, *P* = 0.21	242 men undergoing radical prostatectomy for prostate cancer 149 (62 %) who declined participation, had received preoperative androgen deprivation/radiation therapy, were too thin for harvesting adipose, had metastatic lymph nodes, or had specimens not processed due to technical issues (# in each category NR) Median dioxin-like toxic equivalency level = 22.3 pg/g fat (mean = 25.6) in 37 men with Agent Orange exposure, 15.0 pg/g fat (mean = 16.2) in 56 men without (*P* < 0.001) 27 (73 %) of 37 exposed cases with high (above median) dioxin-like toxic equivalency level versus 20 (36 %) of 56 unexposed cases (*P* < 0.001) Neither Agent Orange exposure nor dioxin-level toxic equivalency level was associated with biochemical recurrence
Li et al. [98]	See above	See above	See above	See above	*47 (51* *%) high-dioxin cases* 4 PSA ≥10 ng/mL 9 pathological stage T3 or T4 31 biopsy Gleason score ≥7 27 prostatectomy Gleason score ≥7 14 adverse pathology 8 positive margins 11 extracapsular extension 2 seminal vesicle invasion 3 lymph node metastasis 4 dead *46 (49* *%) low-dioxin cases* 10 PSA ≥10 ng/mL 5 pathological stage T3 or T4 23 biopsy Gleason score ≥7 26 prostatectomy Gleason score ≥7 19 adverse pathology 17 positive margins 6 extracapsular extension 3 seminal vesicle invasion 0 lymph node metastasis 2 dead	*High versus low dioxin-like toxic equivalency level* Age: 59 versus 57 years, *P* = 0.03 Black race: 43 versus 65 %, *P* = 0.02 Prostate volume: 45 versus 45 cc, *P* = 0.85 PSA ≥10: 9 versus 22 %, *P* = 0.08 Pathological T3/T4: 19 versus 11 %, *P* = 0.26 Biopsy Gleason score ≥7: 66 versus 50 %, *P* = 0.27 Pathological Gleason score ≥7: 57 versus 56 %, *P* = 0.11 Adverse pathology: 30 versus 41 %, *P* = 0.24 Positive margins: 17 versus 37 %, *P* = 0.03 Extracapsular extension: 23 versus 13 %, *P* = 0.19 Seminal vesicle invasion: 4 versus 6 %, *P* = 0.67 Lymph node metastasis: 6 versus 0 %, *P* = 0.24 Death: 8 versus 4 %, *P* = 0.67	See above
Shah et al. [99]	1,495 veterans with prostate cancer treated with radical prostatectomy between 1988 and 2007 at VA health care facilities in West Los Angeles and Palo Alto, California, Augusta, Georgia, and Durham, North Carolina	Period of exposure NR Radical prostatectomy in 1998–2007	Clinical and pathological characteristics of prostate cancer treated with radical prostatectomy Biochemical progression and PSA doubling time after recurrence not shown here	Self-reported Agent Orange exposure as routinely collected by VA health care facilities, abstracted from the electronic medical records system	*206 (14* *%) exposed cases* 50 pathological Gleason score ≥7 104 positive surgical margins 54 extracapsular extension 17 seminal vesicle invasion *1,289 (86* *%) unexposed cases* 288 pathological Gleason score ≥7 642 positive margins 307 extracapsular invasion 144 seminal vesicle invasion	OR for high-grade versus lower-grade disease = 1.33 (0.86–2.06) OR for positive versus negative surgical margins = 1.15 (0.81–1.64) OR for extracapsular extension versus none = 1.17 (0.78–1.74) OR for seminal vesicle invasion versus none = 1.00 (0.51–1.94)	1,747 patients with radical prostatectomy 155 without exposure status 94 of non-white, non-black race 3 of missing race *Exposed versus unexposed cases* mean age = 58.8 versus 62.0 years; *P* < 0.001 mean year of surgery = 2003 versus 1999; *P* < 0.001 56 versus 44 % black; *P* = 0.001 16 versus 8 % ≥35.0 kg/m^2^; *P* = 0.001 mean PSA = 8.4 versus 10.3 ng/mL; *P* = 0.001 median PSA = 6.1 versus 7.4 ng/mL; *P* = 0.001 30 versus 50 % clinical stage T2–T3; *P* < 0.001 16 versus 16 % biopsy Gleason score ≥7; *P* = 0.84 25 versus 23 % pathological Gleason score ≥7; *P* = 0.66 48 versus 49 % positive margins; *P* = 0.86 27 versus 25 % extracapsular extension; *P* = 0.50 8 versus 12 % seminal vesicle invasion; *P* = 0.20 1 versus 2 % lymph node metastasis; *P* = 0.99 ORs adjusted for preoperative PSA level, age at surgery, biopsy Gleason score, year of surgery, clinical stage, race, and center location; results similar after stratification by race Agent Orange exposure was positively associated with biochemical (PSA-based) recurrence and shorter PSA doubling time after recurrence; similar results in a low-risk subset reported in Kane et al. [58]

**Table 2:** Epidemiologic studies of Vietnam veterans without estimated Agent Orange/TCDD exposure

Author	Study subjects	Period of exposure and follow-up	Definition of outcome	Definition of exposure	Level of exposure (with N cases)	RR (95 % CI)	Comments
*Australian Vietnam veterans*							
Fett et al. [107]	19,205 Australian male National Service conscripts (selected by an initial, random ballot among all men turning 20 years of age, followed by medical and psychologic assessments) who served in the Australian Army in Vietnam, compared with 25,677 Australian male National Service conscripts who served only in Australia; results reported separately for those who served <12 or ≥12 months (latter shown here)	Active service during the Vietnam War (June 1, 1965, through February 28, 1971) Mortality follow-up from discharge date or 2 years after enlistment date until January 1, 1982	Genitourinary cancer mortality, with vital status ascertained from Army records, electoral records, death registers, driver’s license registers, immigration records, police and corrective services records, and welfare and credit records; cause of death classified and coded based on death certificate and available records from hospitals, doctors, coroners, police, Australian Army, courts, governments, and other sources assessed independently by five physicians; estimated 4.3 % of deaths missed in both groups	Active Army service in Vietnam between June 1965 and February 1971	Vietnam veterans: 3 deaths Non-Vietnam veterans: 6 deaths	RR = 0.7 (0.2–3)	Methods and dates of follow-up from Fett et al. [107]
Crane et al. [110]	59,036 male (and 484 female) veterans of the Australian Army, Navy, and Air Force and some civilian personnel (i.e., members of medical and surgical teams, philanthropic organizations, Australian Forces Overseas Fund, official entertainers, and war correspondents) who served on Vietnamese land or in Vietnamese waters for ≥1 day during the period of the Vietnam War (“most” aged 20–40 years) External comparisons with Australian national mortality rates, standardized by age, sex, and calendar year	Active service during the Vietnam War Mortality follow-up from end of service in Vietnam through December 31, 1994; follow-up ≈22–32 years	Prostate cancer mortality ascertained from Department of Defense records, VA records, National Death Index (available from 1980 onward), Electoral Commission rolls, and Health Insurance Commission Medicare database; 3.1 % with unknown vital status, with two-thirds of those with missing data estimated to have emigrated from Australia	Active service on Vietnamese land or in Vietnamese waters for ≥1 da y during the Vietnam War	Vietnam veterans: # of deaths NR	SMR = 1.53 (1.07–2.12) Ratio of SMR for prostate cancer to SMR for all other cancer sites combined = 1.28 (0.91–1.79) Ratio of SMR for prostate cancer to SMR for all other causes of death combined = 1.43 (1.02–2.00)	–
Australian Department of Veterans Affairs [114] Australian Institute of Health and Welfare [115]	40,030 male veterans of the Australian Army, Navy, Air Force, and Citizen Military Forces and some civilian personnel (see Crane et al. [110]) who served on Vietnamese land or in Vietnamese waters for ≥1 day between May 23, 1962, and July 1, 1973 External comparisons with age-standardized expected counts from the Australian Cancer Registry	Active service in Vietnam between May 23, 1962, and July 1, 1973 Cross-sectional health survey in 1996	Prostate cancer diagnosed by a doctor since the first day of service in Vietnam, self-reported on a mailed health questionnaire 422 reported cases contacted for validation study; 316 (75 %) responses received; 201 validated based on information from clinicians, National Death Index, National Cancer Statistics Clearing House, Department of Veterans Affairs database, and documentation provided by the veteran; 105 not validated; 17 not able to be validated; 101 with no response (numbers do not add up to 422)	Active service on Vietnamese land or in Vietnamese waters for ≥1 day during the Vietnam War (between May 23, 1962, and July 1, 1973)	Vietnam veterans: 428 reported cases (1 %) Vietnam veterans: 212 estimated validated cases Expected: 147 cases (95 % CI 123–171)	NR Prevalence ratio (self-reported) = 2.9 (2.5–3.5) Prevalence ratio (estimated validated) = 1.4 (1.2–1.7)	Of 49,944 male veterans to whom a questionnaire was mailed, 40,030 (80 %) completed the questionnaire Authors commented: “It is possible that some misreporting of benign prostatic hypertrophy as prostatic cancer has occurred.” Noncancerous disease of the prostate was reported by 2,970 Vietnam veterans (7 %) versus 9,141 cases expected; prevalence ratio = 0.32 (0.26–0.44) 5 deaths from prostate cancer and 2 incident cases in additional veterans (not yet validated) occurred between the morbidity study [114] and the validation study [115]
Wilson et al. [105]	57,864 male military veterans of the Australian Army, Navy, Air Force, and Citizen Military Forces (excluding civilians; mean age at first service = 23.9 years) who served in Vietnam between May 23, 1962, and July 1, 1973, and were not deceased prior to 1982 Comparisons with Australian male national cancer incidence rates standardized by age and calendar year	Cancer incidence follow-up from 1982 through December 31, 2000 (mean follow-up = 18 years) See Crane et al. [110] for exposure period	Prostate cancer ascertained by the Australian Institute of Health and Welfare by matching with the Australian National Cancer Statistics Clearing House	Active military service in Vietnam during the Vietnam War (May 23, 1962, to July 1, 1973)	Vietnam veterans: 692 cases (15 % of all cancers) Expected: 553 cases (14 % of all cancers expected) Navy Vietnam veterans: 137 cases Army Vietnam veterans: 451 cases Air Force Vietnam veterans: 104 cases	All veterans SIR = 1.25 (1.16–1.34) excluding those with unknown vital status at end All veterans SIR = 1.21 (1.12–1.31) including unknowns Navy SIR = 1.19 (0.99–1.39) excluding unknowns Navy SIR = 1.15 (0.95–1.34) including unknowns Army SIR = 1.27 (1.15–1.38) excluding unknowns Army SIR = 1.23 (1.12–1.35) including unknowns Air Force SIR = 1.28 (1.03–1.52) excluding unknowns Air Force SIR = 1.24 (1.00–1.47) including unknowns	59,179 eligible male Vietnam veterans 1,315 deceased prior to start of study 1,037 with unknown vital status at start of study period; 1,454 with unknown vital status at end of study period 15,041 Australian male military veterans of the Korean War had a significant excess of prostate cancer incidence between 1982 and 1999, based on 731 cases (21 % of all cancers) versus 619 expected excluding those with unknown vital status, SIR = 1.18 (1.09–1.27); and versus 678 expected including those with unknown status, SIR = 1.08 (1.00–1.16). Navy SIRs = 1.30 (1.15–1.46) and 1.20 (1.06–1.35), respectively; Army SIRs = 1.08 (0.98–1.19) and 0.97 (0.88–1.07), respectively; Air Force SIRs = 1.30 (1.00–1.61) and 1.25 (0.96–1.55), respectively (Australian Institute of Health and Welfare 111)
Wilson et al. [112]	59,179 male military veterans of the Australian Army, Navy, Air Force, and Citizen Military Forces (excluding civilians; mean age at first service = 23.9 years) who served in Vietnam between May 23, 1962, and July 1, 1973 Comparisons with Australian male national mortality rates standarized by age and calendar year	Mortality follow-up from completion of Vietnam service through December 31, 2001 (~30–40 years) See Crane et al. [110] for exposure period	Prostate cancer mortality; vital status ascertained for 97.3 % of cohort (2.7 % lost to follow-up) from matching to Department of Defense database, Australian State and Territories Registries of Births, Deaths and Marriages, National Death Index, Veterans’ Affairs Client Data Base, Health Insurance Commission Medicare database, National Cancer Statistics Clearing House, Electoral Commission rolls, and Department of Immigration, Multicultural and Indigenous Affairs database; cause of death determined for 97.7 % of decedents from Registries of Births, Deaths and Marriages and National Death Index	Active military service in Vietnam during the Vietnam War (May 23, 1962, to July 1, 1973)	Vietnam veterans: 107 deaths, 87 expected excluding unknowns, 90 expected including unknowns Navy Vietnam veterans: 22 deaths, 17 expected excluding unknowns, 18 expected including unknowns Army Vietnam veterans: 65 deaths, 56 expected excluding unknowns, 57 expected including unknowns Air Force Vietnam veterans: 19 deaths, 14 expected excluding unknowns, 15 expected including unknowns 1963–1979: 1 death, 1 expected 1980–1990: 11 deaths, 13 expected 1991–2001: 94 deaths, 73 expected	All veterans SMR = 1.23 (0.99–1.46) excluding those with unknown vital status at end All veterans SMR = 1.18 (0.96–1.41) including unknowns Navy SMR = 1.29 (0.75–1.82) excluding unknowns Navy SMR = 1.23 (0.72–1.74) including unknowns Army SMR = 1.17 (0.89–1.46) excluding unknowns Army SMR = 1.14 (0.86–1.41) including unknowns Air Force SMR = 1.38 (0.81–2.10) excluding unknowns Air Force SMR = 1.32 (0.78–2.02) including unknowns 1963–1979 SMR = 0.77 (0.02–4.05) excluding unknowns 1980–1990 SMR = 0.89 (0.44–1.57) excluding unknowns 1991–2001 SMR = 1.29 (1.03–1.55) excluding unknowns	Mean time served in Vietnam = 266 days; 90 % served ≤385 days; 1.5 % served >2 years Among male veterans of the Australian Army who served in Vietnam between 1962 and July 1973, prostate cancer incidence did not differ significantly between the 23,262 veterans who consumed Dapsone for malaria prophylaxis and the 16,945 who did not consume Dapsone (168 and 241 cases, respectively; RR = 0.86, 95 % CI 0.70–1.05), nor did prostate cancer mortality differ significantly between the groups (25 and 31 deaths, respectively; RR = 1.21, 95 % CI 0.69–2.12) (Wilson et al. 113)
Wilson et al. [108]	19,240 Australian male National Service conscripts (selected by an initial, random ballot among all men turning 20 years of age, followed by medical and psychologic assessments) who served in the Australian Army in Vietnam between 1966 and July 1973 (mean age at end of Vietnam war = 26.3 years), compared with 24,729 Australian male National Service conscripts who served only in Australia (mean age at end of Vietnam War = 26.1 years) External comparisons with Australian male national mortality and cancer incidence rates, standardized by age and calendar year	Active service between 1966 and July 1973 Cancer incidence follow-up from 1982 through December 31, 2000 Mortality follow-up from completion of Vietnam service through December 31, 2001	Prostate cancer incidence or mortality ascertained by methods described in Wilson et al. [112, 116] Vital status ascertained for 96.7 % of cohort (2.2 % of Vietnam veterans and 4.1 % of non-Vietnam veterans lost to follow-up); cause of death determined for 98.9 % of decedents	Active military service (by conscription) in Vietnam between 1966 and July 1973	*All National Service conscripts (Vietnam and non-Vietnam)* 144 cases, 122 expected excluding unknowns, 125 expected including unknowns 5 deaths, 12 expected excluding of including unknowns *Vietnam conscripts* 65 cases, 54 expected excluding unknowns, 55 expected including unknowns 0 deaths, 5 expected excluding or including unknowns *Non-Vietnam conscripts* 79 cases, 68 expected excluding unknowns, 70 expected including unknowns 5 deaths, 6 expected excluding unknowns, 7 expected including unknowns	*All conscripts* SIR = 1.18 (0.99–1.38) excluding those with unknown vital status at end SIR = 1.15 (0.96–1.34) including unknowns SMR = 0.44 (0.14–1.01) excluding unknowns SMR = 0.43 (0.14–0.98) including unknowns *Vietnam conscripts* SIR = 1.20 (0.91–1.50) excluding unknowns SIR = 1.18 (0.89–1.47) including unknowns SMR = 0.00 (0.00–0.73) excluding unknowns SMR = 0.00 (0.00–0.71) including unknowns *Non-Vietnam conscripts* SIR = 1.17 (0.91–1.43) excluding unknowns SIR = 1.13 (0.88–1.37) including unknowns SMR = 0.78 (0.25–1.81) excluding unknowns SMR = 0.75 (0.24–1.74) including unknowns	Increase of 291 Vietnam veterans compared with Fett et al. [107] is due to “improvements and amendments to the Nominal Roll of Vietnam Veterans since 1997”; increase of 83 non-Vietnam veterans is due to inclusion of those who died before 1982 and exclusion of those with insufficient personal information Mean time served in Vietnam = 9.2 months; 98 % served ≤13 months in Vietnam
Wilson et al. [108]	See above	See above	See above	See above	*Vietnam conscripts* 65 cases, 0 deaths *Non-Vietnam conscripts* 79 cases, 5 deaths	Vietnam versus non-Vietnam, incidence: RR = 1.05 (0.75–1.48) Vietnam versus non-Vietnam, mortality: RR = 0.00 (0.00–1.41)	See above
O’Toole et al. [109]	450 subjects out of 1,000 randomly selected from 57,643 male veterans of the Australian Army posted to Vietnam; subjects completed a first wave of health interviews between July 1990 and February 1993 (mean = 21.96 years after first return to Australia) and a second wave between April 2005 and November 2006 (mean = 36.10 years after first return to Australia) Comparisons with Australian male national age-specific cancer prevalence data	Deployment to Vietnam between 1962 and 1972 Cross-sectional survey in 2005–2006	Prostate cancer prevalence ascertained by a standardized questionnaire (based on the 2004–2005 Australian Bureau of Statistics National Health Survey) administrated by trained clinical and research interviewers	Active Army service in Vietnam during the Vietnam War	Vietnam Veterans: 1.6 % prevalence	Prevalence ratio = 1.29 (0.34–6.73)	Wave 1: 641 participants (87.0 % of locatable veterans, 67.5 % of those not known to have died) Wave 2: 450 participants (79.4 % of locatable veterans, 51.4 % of those not known to have died) Waves 1 and 2: 391 participants
*Vietnam Experience Study*							
Boyle et al. [101]	Male U.S. Army veterans who first entered military service between January 1, 1965, and December 31, 1971, completed ≥16 weeks of active service time, held a military occupational specialty other than “duty soldier” or “trainee,” served a single term of enlistment, and were discharged in the enlisted pay grades E-1 to E-5, randomly selected from computerized lists, including 9,324 Vietnam veterans (mean age at entry into service = 20.3 years) and 8,989 Vietnam-era veterans who served in Korea, Germany, or the U.S. (mean age at entry into service = 20.5 years)	Active service in Vietnam between January 1, 1965, and December 31, 1971 Mortality follow-up from discharge from active duty through December 31, 1983; mean follow-up = 13.7 years for Vietnam veterans, 13.5 years for non-Vietnam veterans	Prostate cancer mortality ascertained from linkages to the VA Beneficiary Identification Records Locator Subsystem, Social Security Administration, Internal Revenue Service, and National Death Index, and personal contact with next-of-kin; cause of death coded from death certificate by a nosologist blinded to service location; cause of death independently assigned by a blinded medical review panel based on hospital records, autopsy reports, private physician contacts, coroner or medical examiner records, and law enforcement files; cause of death assigned to 93.6 % of Vietnam cohort and 91.9 % of non-Vietnam cohort, assumed alive if unknown	Active Army service in Vietnam between 1965 and 1971	Vietnam veterans: 0 deaths Non-Vietnam veterans: 0 deaths	NR	~4.9 million Vietnam-era Army personnel records 48,513 randomly selected 1,355 records not found 28,577 not qualified 18,581 (9,558 with Vietnam service, 9,023 with non-Vietnam service) qualified for study 268 (234 with Vietnam service, 34 with non-Vietnam service) deaths on active duty Mean time on active duty = 26.1 months for Vietnam veterans, 25.3 months for non-Vietnam veterans Medical panel recategorized 2 of 25 deaths attributed to neoplasm by death certificate, and cited neoplasm as underlying cause of death for 2 deaths attributed to a nonneoplastic cause by death certificate; none were prostate neoplasms
Boehmer et al. [117]	See Boyle et al. [101]; mean age at end of study = 53 years	Mortality follow-up from discharge from active duty through December 31, 2000; mean follow-up = 29.8 years See Boyle et al. [101] for exposure period	Prostate cancer mortality ascertained from linkages to VA Beneficiary Identification Records Locator Subsystem, Social Security Administration Death Master File, and National Death Index Plus; cause of death obtained (for 98.3 % of 1,138 newly identified deaths plus 1 of 9 previously identified deaths with missing cause of death) from National Death Index Plus for deaths between 1984 and 2000, and from death certificates for other deaths; unknown vital status assumed to be living	See Boyle et al. [101]	Vietnam veterans: 1 death, 0.4 per 100,000 person-years Non-Vietnam veterans: 3 deaths, 1.1 per 100,000 person-years	NR	67 % of deaths confirmed by all 3 vital status sources, 29 % by 2 sources, 4 % by 1 source
*Other case*–*control and cohort studies*							
Anderson et al. [122]	SMR study: 43,398 Wisconsin Vietnam veterans and 78,940 Wisconsin non-Vietnam veterans (mean age at discharge = 23 years; mean age at end of follow-up = 37.7 years for Vietnam veterans, 37.9 years for non-Vietnam veterans) drafted in Wisconsin and with permanent residence in Wisconsin, with active service for ≥180 days between January 1, 1964, and December 31, 1975, and alive at discharge during that period; identified from the Wisconsin VA DD214 military service separation file External comparisons to mortality rates for U.S. overall, Wisconsin overall, Wisconsin civilians, and Wisconsin veterans	Military service between January 1, 1964, and December 31, 1975 Mortality follow-up from date of discharge (average year = 1969) through December 31, 1984 (average follow-up = 13 years)	Prostate cancer mortality, with vital status ascertained from Wisconsin Department of Revenue files, Wisconsin Department of Transportation files, and VA Beneficiary Identification Records Locator Subsystem, excluding those with unknown vital status or missing death certificate (5.6 % of Vietnam veterans, 10.1 % of non-Vietnam veterans)	≥6 months of active military service including time in Vietnam between January 1, 1964, and December 31, 1975; Vietnam versus non-Vietnam service classified primarily based on the Wisconsin VA DD214 military service separation file	Vietnam veterans: 0 deaths; expected NR Non-Vietnam veterans: 1 death; expected NR Vietnam-era veterans (with and without service in Vietnam): 1 death; expected NR	SMRs = NR	Anderson et al. [122] also conducted a PMR study; see below
Leavy et al. [118]	606 cases of incident, histologically-confirmed prostate cancer aged 40–75 years at diagnosis, identified from the Cancer Registry of Western Australia, and 471 controls without prostate cancer aged 40–75 years, frequency-matched to cases on age, randomly selected from the Electoral Roll of Western Australia	Exposure period NR Cases diagnosed between January 1, 2001, and August 30, 2002	Prostate cancer, histologically confirmed and recorded in the Cancer Registry of Western Australia, where cancer reporting by pathologists is mandatory	Self-reported Vietnam military service assessed by self-administered questionnaire	*Cases*: 25 (4.5 %) served in Vietnam 535 (95.5 %) did not 4 (0.7 %) served in Korea 556 (99.3 %) did not 43 (7.7 %) served in Southeast Asia 517 (92.3 %) did not *Controls* 7 (1.6 %) served in Vietnam 443 (98.4 %) did not 8 (1.6 %) served in Korea 442 (98.2 %) did not 31 (6.9 % served in Southeast Asia) 419 (93.1 %) did not	OR = 2.12 (0.88–5.06) OR = 2.08 (0.87–5.01), also adjusting for family history OR for prostate cancer with Gleason score ≥7: OR = 2.09 (0.79–5.49)	ORs adjusted for age Served in military: OR = 1.14 (0.88–1.48) Served in area of conflict: OR = 1.05 (0.67–1.64) Served in Korea: OR = 0.48 (0.14–1.63) Served in Southeast Asia: OR = 1.01 (0.62–1.66) Case participation rate = 685 (64 %) of 1,066 invited (of 1,226 identified, among whom those without contact information or physicial refusal to contact were excluded) Control participation rate = 547 (43 %) of 1,272
McBride et al. [119]	2,783 male New Zealand veterans with military service in Vietnam between 1964 and 1972, identified from pay records into the VA New Zealand Vietnam veterans’ database Comparisons with New Zealand male national mortality and cancer incidence rates standardized by age and calendar period	Military service in Vietnam between 1964 and 1972 Cancer incidence and mortality follow-up from January 1, 1988, through December 31, 2008	Prostate cancer incidence ascertained from linkage to New Zealand Ministry of Health database Prostate cancer mortality ascertained from the Mortality Collection database, which records official underlying cause of death based on the registry of Births, Deaths and Marriages New Zealand, traffic accident reports, coroners’ inquiries, hospital diagnoses, pathology records, and cancer registry entries	Military service in Vietnam between 1964 and 1972; New Zealand records on Vietnam veterans regarded as “complete”	Vietnam veterans: 136 cases, 13 deaths Expected: 116.2 cases, 12.6 deaths	SIR = 1.17 (0.98–1.39) SMR = 1.03 (0.55–1.76)	Original cohort of 3,361 male Vietnam veterans 36 deaths during Vietnam service 3 additional deaths before 1988 539 not matched to health data (57 % with no address, 30 % with an overseas address, 6 % with unconfirmed death)
Yi et al. [120]	185,265 Korean veterans of the Vietnam War identified by the Ministry of National Defense and Ministry of Government Administration and Home Affairs as of June 2004 Comparisons with Korean male national cancer incidence rates, standardized by age and calendar year	Military service in Vietnam between 1964 and 1973 Cancer incidence follow-up from January 1, 1992, through December 31, 2003	Prostate cancer incidence identified from the Korean National Cancer Incidence Database	Military service in Vietnam during Korean involvement between 1964 and 1973	All Vietnam veterans: 125 cases (102.3 expected) Enlisted soldiers: 48 cases Noncommissioned officers: 18 cases Officers: 59 cases 1992–1997: 17 cases 1998–2003: 108 cases	All Vietnam veterans: SIR = 1.22 (1.02–1.46) Enlisted soldiers: SIR = 0.85 (0.64–1.14) Noncommissioned officers: SIR = 0.81 (0.51–1.29) Officers: SIR = 2.49 (1.93–3.21) 1992–1997: SIR = 1.27 (0.78–2.08) 1998–2003: SIR = 1.21 (1.00–1.48)	187,897 veterans identified 2,137 deceased or emigrated before 1992 495 diagnosed with cancer before 1992 *Serum TCDD levels (pg/g lipid) in Yi et al.* [97] Enlisted soldiers (N = 66): mean = 1.3, median = 0.9, geometric mean ± SD = 1.0 ± 1.8, range 0.3–11.4, 25th–75th pctl = 0.7–1.2 Noncommissioned officers (N = 16): mean = 1.1, median = 1.0, geometric mean ± SD = 0.9 ± 2.0, range 0.2–2.7, 25th–75th pctl = 0.8–1.1 Officers (N = 20): mean = 1.2, median = 0.9, geometric mean ± SD = 1.0 ± 1.7, range 0.6–4.0, 25th–75th pctl = 0.8–1.2 *P* value for one-way analysis of variance = 0.84
*Proportionate mortality and morbidity studies*							
Lawrence et al. [121]	555 deceased male Vietnam veterans and 941 deceased male non-Vietnam veterans discharged between July 1, 1970, and June 30, 1973, who died in New York State between 1970 and 1980	Military discharge between July 1, 1970, and June 30, 1973 Death between 1970 and 1980	Genitourinary cancer mortality, with vital status ascertained from the VA Beneficiary Identification Records Locator Subsystem and cause of death ascertained from New York State Vital Records and other death certificates	Military service in Vietnam, with information obtained from Defense Manpower Data Center and, for 113 Army personnel discharged in 1971, directly from personnel records	Vietnam veterans: 5 deaths Non-Vietnam veterans: 10 deaths	Mortality OR = 0.82 (0.28–2.45)	Mortality ORs adjusted for age, race, and education Deaths due to motor vehicle accidents, nonmotor vehicular injuries of transport, other accidents and burns, and liver disease including cirrhosis were removed from the reference category of other causes of death In a comparison of Vietnam-era veterans versus nonveterans who died in New York State between 1965–1967 and 1970–1980, the mortality OR for genitourinary cancer in veterans versus nonveterans was 1.19 (0.82–1.71), adjusted for age and race, based on 37 veteran deaths and 144 nonveteran deaths
Anderson et al. [122]	PMR study: 923 deceased white male Vietnam veterans and 1,571 deceased white male non-Vietnam veterans who served for ≥6 months between 1964 and 1975 (and 109,225 other deceased white male veterans), were discharged alive from military service, and died in Wisconsin between 1964 and July 1983; identified from the Wisconsin VA grave registration file	Military service between January 1, 1964, and December 31, 1975 Deaths between 1964 and July 1983	Prostate cancer mortality ascertained from the Wisconsin Department of Health and Social Services Death Index Tape for 1960–1978, with back-up from the VA Beneficiary Identification Records Locator Subsystem for vital status	≥6 months of active military service including time in Vietnam between January 1, 1964, and December 31, 1975; Vietnam versus non-Vietnam service classified primarily based on the Wisconsin VA DD214 military service separation file	Vietnam veterans: 0 deaths, NR expected Non-Vietnam Vietnam-era veterans: 2 deaths, NR expected All other (non-Vietnam-era) state veterans: 1,763 deaths, 1,859.01 expected Vietnam-era veterans (with and without service in Vietnam): 2 deaths, NR expected	Vietnam veterans versus nonveterans: PMR = NR (0) Non-Vietnam veterans versus nonveterans: PMR = NR All other veterans versus nonveterans: PMR = 0.95 (0.91–0.99) Vietnam-era veterans versus nonveterans: PMR = NR	4,441 deaths identified 1,734 ineligible 22 missing discharge record or death certificate 99 black males, 45 American Indian males, 6 Hispanic males, 12 males of unknown race, and 29 females
Holmes et al. [123]	Deceased West Virginia male veterans, including 615 Vietnam and 610 non-Vietnam veterans (average age at death = 35.4 and 35.1 years, respectively), who served in the military for ≥90 days between August 1, 1964, and March 28, 1973, resided in West Virginia for ≥6 months prior to entry into active service, and were honorably discharged, identified from a list of applicants for a military bonus offered by the state VA department, linked to state mortality listing by name (1968–1978) or Social Security number (1979–1983)	Active military service between August 1, 1964, and March 28, 1973 Death between 1968 and 1983	Male genital organ cancer mortality identified from complete listing of West Virginia resident deaths from state Health Statistics Center	Military service in Vietnam based on state military bonus file, confirmed by review of DD214 discharge form	Vietnam veterans: 4 deaths Non-Vietnam veterans: 3 deaths	Vietnam veterans versus non-Vietnam veterans: PMR = 2.22 (0.62–5.79) Vietnam veterans versus non-veterans: PMR = 1.82 (0.49–4.61) Non-Vietnam veterans versus non-veterans: PMR = 0.83 (0.18–2.54) All veterans versus non-veterans: PMR = 1.23 (0.50–2.54)	PMRs standardized by age and year of death; 95 % white race
Breslin et al. [127]	Deceased male ground troops who served in the U.S. Army or Marine Corps between July 4, 1965, and March 1, 1973, and were reported to be deceased outside of military action as of July 1, 1982, including 24,235 who served in Vietnam and 26,685 who did not serve in Southeast Asia (>50 % deaths at ages 25–34 years; 0.74 % of Vietnam and 2.6 % of non-Vietnam veterans’ deaths at >65 years)	Active military service between July 4, 1965, and March 1, 1973 Death between July 4, 1965, and July 1, 1982	Prostate cancer mortality, with vital status ascertained from the VA Beneficiary Identification Records Locator Subsystem and cause of death coded by blinded nosologists from death certificates available from VA files (for ~70 of decedents) or state of last known residence (for ~27 % of decedents), and from casualty reports issued by the Department of Defense (for 3.1 % of decedents); cause of death ascertained for 98.4 % of decedents	Service in U.S. Army or Marine Corps in Vietnam between July 4, 1965, and March 1, 1973	Army Vietnam veterans: 30 deaths Marine Corps Vietnam veterans: 5 deaths Deaths in non-Vietnam veterans NR	Army PMR = 0.92 (0.55–1.23) Marine Corps PMR = 1.29 (0.16–10.3)	186,000 deceased Army or Marine Corps Vietnam-era veterans 75,617 names randomly selected 22,332 ineligible 1,032 with indeterminate eligibility (military records not found) 832 with unknown cause of death 501 who served outside Vietnam in Southeast Asia or had unknown place of service in Southeast Asia PMRs standardized by age and race
Clapp et al. [125]	Massachusetts male veterans diagnosed with cancer between January 1, 1982, and August 1988, including 230 patients (214 aged 30–59 at diagnosis) who received a Vietnam bonus and 939 others (727 aged 30–59 years at diagnosis) who received a Vietnam-era bonus from the State of Massachusetts Comparison group: all eligible patients with cancer other than soft tissue sarcoma, non-Hodgkin lymphoma, kidney cancer, and the cancer of interest (i.e., prostate)	Military service during the Vietnam era Cancer diagnosis between January 1, 1982, and August 1988	Prostate cancer identified from the Massachusetts Cancer Registry	Military service in Vietnam, i.e., identification by the Massachusetts Office of the Commissioner of Veterans Services as having received a state Vietnam bonus (requiring proof of service, usually the DD214 discharge form)	Vietnam veterans: 8 cases (3 % of 234 cancers among 230 subjects) at all ages Other Vietnam-era veterans: 66 cases (7 % of 961 cancers among 939 subjects) at all ages	Standardized morbidity OR = NR	A military archivist estimated that 90–95 % of all eligible Massachusetts residents received the Vietnam and Vietnam-era bonuses, mostly during the mid-1970s Numbers of total cancers (234 in Vietnam veterans, 961 in other Vietnam-era veterans) differ from numbers of subjects (230 Vietnam veterans, 939 Vietnam-era veterans) and may include multiple primary cancers
Visintainer et al. [124]	Deceased Michigan male veterans (3,364 who served in Vietnam and 5,229 who served outside Vietnam) identified from the Michigan Department of Management and Budget’s Vietnam-era Bonus List, which required ≥190 days of honorable service, ≥6 months of residence in Michigan, a certified copy of discharge from military service, not being listed as missing in action or deceased during period of service, and application for bonus between December 23, 1974, and June 30, 1980; aged 18–29 years during period of service, aged 35–54 years at death	Military service between January 1, 1961, and September 1, 1973 Death between 1974 and 1989	Male genital system cancer mortality, with vital status and cause of death determined by linkage to the Michigan Department of Public Health death certificate database	Military service in Vietnam between January 1, 1961, and September 1, i.e., identification as an in-country Vietnam veteran on the Michigan Vietnam-era Bonus List	Vietnam veterans: 8 deaths Non-Black Vietnam veterans: 8 deaths Black Vietnam veterans: 0 deaths	All races PMR = 1.06 (0.64–1.65) Non-Black PMR = 1.00 (0.59–1.58) Black PMR = NR (0)	151,377 Vietnam veterans and 225,651 non-Vietnam veterans on Bonus List → 3,701 (2.4 %) Vietnam decedents and 6,026 (2.7 %) non-Vietnam decendents → 3,364 Vietnam and 5,229 non-Vietnam male decendents aged 35–54 years “It is not known if [the Bonus List] is representative of Michigan Vietnam-era veterans.” Denominator for cancer PMRs = total cancer deaths (not total deaths), standardized by age, race, and calendar period
Watanabe et al. [128]	See Breslin et al. [127]; 33,833 deceased male ground troops who served in Vietnam (age at death ≥45 years for 24.7 % of Army and 16.9 % of Marine Corps personnel) and 36,797 who did not serve in Southeast Asia (age at death ≥45 years for 22.5 % of Army and 9.4 % of Marine Corps personnel) External comparisons to U.S. male national mortality distribution	Death between July 4, 1965, and June 30, 1988 See Breslin et al. [127] for exposure period	Prostate cancer mortality, with cause of death available for 97.6 % of decedents; see Breslin et al. [127] for mortality ascertainment methods	See Breslin et al. [127]	Army Vietnam veterans: 58 deaths Army non-Vietnam veterans: 103 deaths Marine Corps Vietnam veterans: 9 deaths Marine Corps non-Vietnam veterans: 6 deaths	Army Vietnam versus Army non-Vietnam: PMR = 0.92 0–10 years since leaving Vietnam: PMR = 0.98 11–15 years since leaving Vietnam: PMR = 0.77 ≥16 years since leaving Vietnam: PMR = 1.12 Army Vietnam versus Army + Marine non-Vietnam: PMR = 0.87 Marine Vietnam versus Marine non-Vietnam: PMR = 0.79 0–10 years since leaving Vietnam: PMR = 1.13 11–15 years since leaving Vietnam: PMR = 1.26 ≥16 years since leaving Vietnam: PMR = 0.54 Marine Vietnam versus Army + Marine non-Vietnam: PMR = 0.90 Army Vietnam versus U.S. men: PMR = 1.08 Army non-Vietnam versus U.S. men: PMR = 1.22, *P* < 0.05 Marine Vietnam versus U.S. men: PMR = 1.17 Marine non-Vietnam versus U.S. men: PMR = 1.30	75,617 names randomly selected from 186,000 deceased Army or Marine Corps Vietnam-era veterans +all 15,038 veterans who died in 1982–1984 +11,851 names randomly selected from 59,259 veterans who died in 1984–1988 26,929 ineligible 2,353 with indeterminate eligibility (military records not found) 1,742 with unknown cause of death and other variables 852 who served outside Vietnam in Southeast Asia PMRs standardized by age at death, race, and calendar year 58 % with length of service = 2–4 years
Clapp et al. [126]	Massachusetts male veterans diagnosed with cancer at ages 35–64 years between 1988 and 1993, including 245 patients who received a Vietnam bonus and 999 others who received a Vietnam-era bonus from the State of Massachusetts Comparison group = all eligible cases with cancer of the gastrointestinal tract (stomach, pancreas, colon, and rectum)	Military service during the Vietnam era Cancer diagnosis between 1988 and 1993	See Clapp et al. [125]	See Clapp et al. [125]	Vietnam veterans: 15 cases (6.1 % of 245 cancers) Other Vietnam-era veterans: 118 cases (11.8 % of 999 cancers)	Standardized morbidity OR = 0.8 (0.4–1.6)	ORs adjusted for age

**Table 3:** Epidemiologic studies of manufacturers and sprayers of herbicides potentially contaminated with TCDD

Author	Study subjects	Period of exposure and follow-up	Definition of outcome	Definition of exposure	Level of exposure (with N cases)	RR (95 % CI)	Comments
Ott and Zober [145]	243 male workers in German plant where an accident entailing releasing of TCDD occurred in 1953, who were involved in clean-up, repair, maintenance, or demolition activities; serum TCDD level available for all cohort members	Employment during 1953–1969 in accident-related activities; follow-up 1953–1992	Prostate cancer mortality from death certificates obtained through health and safety administration Incidence of prostate cancer from medical and hospital records and contacts with cohort members	Ever-employment in accident-related activities Exposure derived from serum TCDD levels: low (<0.1 µg/kg body weight), medium (0.1–0.99 µg/kg body weight), high (≥ 1 µg/kg body weight) 113 workers with chloracne	Exposed: 0 deaths Exposed: 4 incident cases Low exposure: 3 cases Medium exposure: 1 case High exposure: 0 cases	SMR = 0 (0–1.9) SIR = 1.1 (0.3–2.8) SIR = 2.5 (0.5–7.4) SIR = 1.1 (0–5.9) SIR = 0 (0–2.5)	Not included in the pooled analysis [17] No results on prostate cancer reported for chloracne sub-cohort
Kogevinas et al. [17]	21,863 male and female workers exposed to phenoxy herbicides and chlorophenols in manufacture or spraying; pooled analysis of 36 cohorts (32 manufacture, 4 spraying) from 12 countries (Austria, Australia, Canada, Denmark, Finland, Germany, Italy, the Netherlands, New Zealand, Sweden, United Kingdom, United States)	Employment and cancer mortality follow-up period varied by cohort and country; overall they extended during 1939–1992	Prostate cancer mortality from death certificates obtained from national mortality registries in 10 countries, place of residence (Italy) or contact to companies, insurance, physicians and family members (Germany)	Exposure to TCDD defined as (1) employment during manufacture of spraying of 2,4,5-T, PCP or other potentially contaminated compounds, or (2) employment in plants with documented (biomonitoring or environmental measurements) exposure to TCDD Biomonitoring data available for 573 workers from 10 cohorts 13,831 workers (63.3 %) classified as exposed to TCDD	Exposed: 43 deaths Unexposed: 25 deaths	Exposed: SMR = 1.11 (0.81–1.50) Unexposed: SMR = 1.10 (0.71–1.62)	–
Steenland et al. [136]	5,132 male workers employed in 12 U.S. plants manufacturing herbicides contaminated with TCDD	Ever employment during 1942–1984; follow-up 1942–1993	Prostate cancer mortality from death certificates obtained from National Death Index, Social Security Administration, and Internal Revenue Service	Ever employment in the plants Biomonitoring data available for 253 workers 630 workers with history of chloracne	Exposed: 28 deaths	SMR = 1.17 (0.78–1.69)	Included in the pooled analysis [17], with follow-up to 1987 No results on prostate cancer reported for chloracne sub-cohort
‘t Mannetje et al. [140]	1,025 male and female phenoxy herbicide production workers and 703 sprayers in New Zealand	Employment 1969–1984 (production workers; 813 exposed); registration 1973–1984 (sprayers; 699 exposed); follow-up 1969–2000	Prostate cancer mortality from national registry	Employment in herbicide production or spraying	Production: 1 death Spraying: 2 deaths	Production: SMR = 0.37 (0.01–2.08) Sprayers: SMR = 0.60 (0.07–2.16)	Included in the pooled analysis [17] with follow-up to 1986
Collins et al. [137]	773 male PCP manufacture workers with exposure to chlorinated dioxins, including 196 with exposure to TCDD, employed in one U.S. plant; serum TCDD level available for 128 workers	Employent during 1937–1980; period of follow-up unspecified	Prostate cancer mortality from death certificates	Ever-employment in PCP manufacture Estimated cumulative TEQ exposure estimated from serum levels of 5 dioxins and occupational history: low (<0.7 ppb-years), medium (0.7–3.99 ppb-years), high (4.0+ ppb-years)	Whole cohort: 8 deaths Excluding 196 TCP workers: 7 deaths Low exposure: 4 deaths Medium exposure: 2 deaths High exposure: 2 deaths Dose–response (per 1 ppb-year)	SMR = 1.0 (0.4–1.9) SMR = 1.0 (0.4–2.1) SMR = 0.8 (0.1–2.9) SMR = 0.6 (0.1–2.4) SMR = 1.4 (0.4–3.6) RR = 1.002 (0.962–1.045)	Included in the pooled analysis [17] with follow-up to 1987, and in the study by Steenland et al. [136] with follow-up to 1993
Collins et al. [138]	1,615 male TCP manufacture workers with exposure to TCDD employed in one U.S. plant, including 196 PCP production workers; serum TCDD level available for 280 workers	Employment during 1942–1982; follow-up 1942–2003	Prostate cancer mortality from death certificates	Ever-employment in TCP or 2,4,5-T manufacture	Whole cohort: 21 deaths Excluding PCP workers: 20 deaths	SMR = 1.4 (0.9–2.2) SMR = 1.5 (0.9–2.4)	Included in the pooled analysis [17] with follow-up to 1987, and in the study by Steenland et al. [136] with follow-up to 1993
McBride et al. [141]	1,754 male and female production workers in New Zealand	Employment during 1969–2003; follow-up 1969–2004	Prostate cancer mortality from national registry	Employment in herbicide production	Whole cohort: 3 deaths <3 months employment: 3 deaths 3+ months employment: 0 deaths <15 years since last worked: 0 deaths ≥15 years since last worked: 3 deaths Hired pre-1976: 3 deaths Hired post-1975: 0 deaths	SMR = 0.54 (0.11–1.56) SMR = 0.62 (0.13–1.82) SMR = 0.00 (0.00–4.67) SMR = 0.00 (0.00–4.83) SMR = 0.62 (0.13–1.81) SMR = 0.58 (0.12–1.68) SMR = 0.00 (0.00–9.36)	Included in the pooled analysis [17] with follow-up to 1986 and in the study by ‘t Mannetje et al. [140] with follow-up to 2000
McBride et al. [142]	1,599 male and female production workers in New Zealand	Employment during 1969–1988; follow-up 1969–2004	Prostate cancer mortality from national registry	Employment during 2,4,5-T production; 1,134 workers classified as TCDD exposed and 346 as unexposed	Exposed to TCDD: 1 death Unexposed to TCDD: 2 deaths	SMR = 0.2 (0.0–1.2) SMR = 1.9 (0.2–6.7)	Included in the pooled analysis [17] with follow-up to 1986 and in the study by ‘t Mannetje et al. [140] with follow-up to 2000; subset of population studies by McBride et al. [142]
Boers et al. [143, 146]	2,106 male workers in two Dutch plants, one with 2,4,5-T as main product (factory A) and the other with 4-*chloro*-2-methylphenoxyacetic acid, 4-*chloro*-3-methylphenoxy propanoic acid, and 2,4-D as main products (factory B); serum TCDD level available for 187 workers (101 from factory A, 86 from factory B)	Employment 1955–1985 (plant A), 1965–1986 (plant B); follow-up to 2006	Prostate cancer mortality from national registry	Employment in selected departments (or entering them on a regular basis), or exposure to an accident in factory A in 1963	*Factory A* Exposed: 6 deaths Unexposed: 2 deaths *Factory B* Exposed: 4 deaths Unexposed: 2 deaths	Factory A: HR = 2.93 (0.61–14.1) Factory B: HR = 2.68 (0.48–14.9) HR for ln TCDD level = 1.08 (0.79–1.49) in total cohort HR for ln TCDD level = 1.29 (0.85–1.94) in factory A	Included in the pooled analysis [17] with follow-up to 1986; 4 of 6 exposed cases in Factory A exposed to accident
Ruder and Yiin [139]	2,122 workers at 4 U.S. PCP production plants	Employment from 1930s until 1980s, one plant until 2005; follow-up 1940–2005	Prostate cancer mortality from death certificates	Employment in TCP production (exposure to TCDD, N = 720)	Exposed: 8 deaths Unexposed: 18 deaths Whites: 21 deaths Non-Whites: 5 deaths	Exposed: SMR = 1.08 (0.47–2.12) Unexposed: SMR = 1.01 (0.60–1.60) Whole cohort, whites: SMR = 1.04 (0.64–1.59) Whole cohort, non-whites: SMR = 1.00 (0.32–2.33)	Included in the pooled analysis [17] and in the study by Steenland et al. [136]; partial overlap with Collins et al. [138]
Manuwald et al. [144]	1,191 male production workers in Germany	Employment 1952–1984; follow-up 1952–2007	Prostate cancer mortality from death certificates obtained from local population registries	Ever-employment (minimum 3 months) in the plant	Ever employed: 19 deaths	SMR = 1.37 (0.82–2.13)	Included in the pooled analysis [17] with follow-up to 1987

**Table 4:** Epidemiologic studies of the Seveso industrial accident

Author	Study subjects	Period of exposure and follow-up	Definition of outcome	Definition of exposure	Level of exposure (with N cases)	RR (95 % CI)
Consonni et al. [150]	390 men in zone A (“very high contamination”), 3017 in zone B (“high contamination”), 19,199 in zone R (“low contamination”), 139,516 in reference area (“unaffected”) based on their official residence on the day of the industrial accident or at entry (by birth or migration) into the area	Exposure during 1976–1985; follow-up 1976–2001	Mortality from prostate cancer from death certificates	Residence in contaminated area in 1976, migration into the area during 1976–1985 Range of mean TCDD levels in soil (µg/m^2^): Zone A: 15.5–580.4 Zone B: 1.7–4.3 Zone R: 0.9–1.4 Reference: NA Median lipid-adjusted TCDD levels in serum (pg/g or ppt): Zone A: 447.0 in 1976–77, 73.3 in 1992–96 Zone B: 94.0 in 1976–77, 12.4 in 1992–96 Zone R: 48.0 in 1976–77, NA in 1992–96 Reference: NA in 1976–77, 5.5 in 1992–96	Zone A: 1 death Zone B: 8 deaths Zone R: 65 deaths	Zone A RR = 0.87 (0.12–6.17) Zone B RR = 0.88 (0.44–1.77) Zone R RR = 1.06 (0.81–1.38)
Pesatori et al. [151]	352 men in zone A (“very high contamination”), 2,471 in zone B (“high contamination”), 15,715 in zone R (“low contamination”), 88,349 in reference area (“unaffected”) based on their official residence on the day of the industrial accident	Exposure at accident on July 10, 1976; follow-up 1976–1996	Incidence of prostate cancer from cancer registry	Residence in contaminated area in 1976	Zone A: 0 cases Zone B: 7 cases Zone R: 39 cases	Zone A RR = 0 Zone B RR = 0.94 (0.45–1.99) Zone R RR = 0.75 (0.54–1.99)

To provide a visual overview of the results of all studies that reported relative risk (RR) estimates for prostate cancer incidence, prevalence, or mortality in association with Agent Orange/TCDD exposure or surrogates for exposure, forest plots are provided in supplementary online figures. Figure S1 shows the results of epidemiologic studies of veterans with estimated exposure to Agent Orange/TCDD, Figure S2 shows the results of epidemiologic studies of Vietnam veterans without estimated exposure to Agent Orange/TCDD, and Figure S3 shows the results of epidemiologic studies of manufacturers and sprayers of herbicides potentially contaminated with TCDD and studies of the Seveso, Italy, industrial accident. To avoid skewing the data by selectively displaying some results but not others, all potentially relevant RR estimates for overall prostate cancer are shown. Results are not shown for clinical subgroups of prostate cancer. Summary RRs are not calculated to avoid presenting a single non-representative RR from each study, obscuring information on potential exposure–response trends, inappropriately equating disparate exposures such as measured serum TCDD levels and self-reported Agent Orange exposure, and overstating the homogeneity and statistical precision of results [59, 60].

It is important to emphasize that this graphic display of results does not take study quality into account, and it does not more heavily weight studies that used more valid measures of TCDD exposure. Therefore, the results shown in the figures must be interpreted in light of the study descriptions presented in the text and tables.

### Studies of veterans with estimated Agent Orange/TCDD exposure

#### Air Force Health Study

The Air Force Health Study is a prospective matched cohort study with up to 20 years of follow-up on 1,261 veterans of Operation Ranch Hand, the U.S. Air Force program that conducted aerial spraying of herbicides, including Agent Orange, in South Vietnam from 1962 through 1971 [61]. Due to their direct handling of herbicides, Operation Ranch Hand veterans (hereafter referred to as Ranch Hands) had the greatest potential for exposure to Agent Orange/TCDD during the Vietnam War. The unexposed comparison group in this study comprised 19,101 personnel assigned to aerial cargo missions in Southeast Asia during the same time period. Up to 10 comparison subjects (a combined total of 10,133) were initially matched to each Ranch Hand on date of birth, race, rank, and military occupation during their duty in Southeast Asia. Ranch Hands and one living comparison subject from each matched set, with replacement of non-participating comparison subjects, were invited to participate in periodic physical examinations and questionnaires. TCDD levels measured at the 1987 examination established that average serum TCDD levels were higher in Ranch Hands than comparison subjects within all strata of military occupation and overall (Table 5).

**Table 5:** Serum 2,3,7,8-tetrachlorodibenzo-*p*-dioxin levels in Air Force Health Study participants in 1987 (reproduced from Wolfe et al. [66])

Stratum	Ranch Hands			Comparison subjects		
	N	Median (ppt)	Range (ppt)	N	Median (ppt)	Range (ppt)
Flying officers (pilot)	247	7.3	0.0–42.6	239	4.7	0.0–18.5
Flying officers (navigator)	63	9.3	1.1–35.9	53	4.5	2.2–7.9
Nonflying officers	19	6.6	3.1–24.9	11	4.3	0.0–6.0
Flying enlisted personnel	152	17.2	0.0–195.5	137	4.0	0.0–12.7
Nonflying enlisted personnel	407	23.6	0.0–617.7	416	3.9	0.0–54.8
All personnel	888	12.4	0.0–617.7	856	4.2	0.0–54.8

The Air Force Health Study included two components: a mortality study and a morbidity study. The mortality study found no significant difference in prostate cancer mortality between Ranch Hands and comparison subjects as of 1993, although few prostate cancer deaths were observed [standardized mortality ratio (SMR) = 3.18 [0.39–11.5]^1^; 2 deaths [62] (Table 1; Figure S1). With follow-up through 2000, prostate cancer mortality did not differ significantly between Ranch Hands and the general U.S. male population (SMR = 0.70 [0.12–2.33]; 2 deaths) or between comparison subjects and the general U.S. male population (SMR = 0.77 [0.20–2.09]; 3 deaths) [63].

In the morbidity study, health conditions were identified in Ranch Hands and matched comparison subjects through physical examinations in 1982 (baseline), 1985, 1987, 1992, 1997, and 2002 (including digital rectal examination in all years and PSA testing beginning in 1992), questionnaire responses that were validated by review of medical records (which were also used to identify unreported diagnoses, as possible), and death certificates [61]. In a 1999 study, Ranch Hands were classified according to serum TCDD levels measured in 1987 (for most subjects) or 1992, extrapolated to the end of military service in Southeast Asia under the assumption of a constant half-life of 8.7 years, and categorized as “background” (≤10 ppt), “low” (10–≤94 ppt), or “high” (>94 ppt) [64]. As of 1997, the adjusted odds ratio (OR) for prostate cancer in Ranch Hands versus comparison subjects (33 cases) was 0.4 (0.1–0.8; 7 cases) in those with background serum TCDD levels, 0.6 (0.3–1.5; 8 cases) in those with low serum TCDD levels, and 0.7 (0.2–2.2; 4 cases) in those with high serum TCDD levels, with no apparent dose–response trend (*P* = 0.41) (Table 1; Figure S1).

In a reanalysis of this dataset, Kayajanian [65] reclassified TCDD exposure according to a body burden model and—in a departure from the original study design—compared observed prostate cancer incidence in all Air Force Health Study participants (including Ranch Hands and comparison subjects) against expected counts based on U.S. national cancer incidence rates. Among white men, the standardized incidence ratio (SIR) was consistently elevated but did not change significantly with higher estimated TCDD body burden, whereas among black men, the SIR was elevated but *decreased* significantly with higher estimated TCDD body burden (*P* < 0.006, <0.004, and <0.002 for various comparisons between higher and lower exposure categories) (Table 1; Figure S1).

The Air Force Health Study was not designed to compare Ranch Hands or comparison subjects with an external group outside of Southeast Asia because the objective of the study was “to determine the effects of exposure to TCDD and not of service in Southeast Asia” [66]. Nevertheless, in a 2004 study, the study investigators deviated from the original study design by comparing cancer incidence in Ranch Hands and comparison subjects with that expected in the U.S. general population [63]. Deviation from the original protocol is inappropriate if the a posteriori analysis is more likely to be biased and especially if it is conducted in an effort to detect significant results. In this case, due to greater health care access among veterans [68], comparisons of veterans with the general population were more susceptible to upward bias due to differences in diagnostic intensity than comparisons between Ranch Hands and designated comparison subjects. Bias due to a “healthy serviceman” effect is not a major concern, given the long latency period and advanced average age at onset of prostate cancer [69]. All results reported for prostate cancer in this study were for white men only.

Prostate cancer incidence as of 1999 was significantly higher than expected among both Ranch Hands (SIR = 1.46 [1.04–2.00]; 36 cases) and comparison subjects (SIR = 1.62 [1.23–2.10]; 54 cases), but no significant difference was detected between the SIRs for Ranch Hands and comparison subjects (*P*_heterogeneity_ = 0.62) (Table 1; Figure S1) [63]. Prostate cancer mortality was not significantly increased above expectation in either study group; thus, the incidence finding was consistent with confounding by diagnostic intensity. Borderline significant differences in prostate cancer incidence between Ranch Hands and comparison subjects were detected only in subgroup analyses that compared Ranch Hands who spent 100 % of their Southeast Asia service in Vietnam (SIR = 1.66 [1.00–2.60]) with comparison subjects who spent 0 % of their Southeast Asia service in Vietnam (SIR = 0.59 [0.15–1.61]; *P*_heterogeneity_ = 0.08), or that were restricted to veterans who spent a maximum of 2 years in Southeast Asia (SIR for Ranch Hands = 1.54 [0.98–2.32]; SIR for comparison subjects = 0.68; *P*_heterogeneity_ = 0.05). The former association may have been influenced by differences in diagnostic intensity if health care usage was greater among Vietnam than non-Vietnam veterans, as suggested by some data [70]. Again, this comparison marked a departure from the original Air Force Health Study design and was more likely to be confounded than contrasts between Ranch Hands and the original comparison subjects. The latter association, which was based on 68 % of Ranch Hands and 47 % of comparison subjects, could have been due to selection bias resulting from conditioning on a characteristic [71]—shorter duration of military service—that was more frequent among Ranch Hands and that could have been associated with greater health care usage if, for example, servicemen served for a shorter time due to health problems requiring medical attention. The finding of a positive association restricted to this subgroup is also counterintuitive, since shorter duration of service among Ranch Hands should have decreased cumulative Agent Orange exposure.

In internal cohort analyses, serum TCDD levels were extrapolated to the end of service in Vietnam assuming a constant half-life of 7.6 years, and categorized as “background” (≤10 ppt), “low” (>10–≤118.5 ppt), or “high” (>118.5 ppt) [63]. After restriction to veterans who spent at most two years in Southeast Asia, the adjusted RR of prostate cancer in Ranch Hands versus comparison subjects increased with higher serum TCDD levels and was significantly elevated for those with high serum TCDD (RR = 6.04 [1.48–24.61]; 5 cases) (Table 1). However, log-transformed serum TCDD level itself was not significantly associated with prostate cancer risk in this subgroup (RR = 1.48 [0.93–2.35]). In another analysis restricted to Ranch Hands who spent 100 % of their Southeast Asia service in Vietnam versus comparison subjects who spent 0 % of their Southeast Asia service in Vietnam, the RR for high serum TCDD was 4.67 (0.75–29.07; 4 cases), with an RR of 1.07 (0.64–1.78) per unit increase in log-transformed serum TCDD level.

The authors noted that 53 of the 90 prostate cancer cases (25 of 36 [69 %] among Ranch Hands and 28 of 54 [52 %] among comparison subjects) were “diagnosed as a direct result of the examinations” scheduled for the study, and that “these data are consistent with the hypothesis that the increased SIR for … prostate cancer might be at least partially explained by the medical examinations that these men received” [63]. These case numbers were sufficient to account for the excess prostate cancer incidence observed among Ranch Hands versus the general population (36 cases observed vs. 24.71 expected) and among comparison subjects versus the general population (54 cases observed vs. 33.34 expected). The results of the internal analyses in selected subgroups were potentially biased and difficult to interpret due to the reasons described above. Due to insufficient information, it is unclear whether discrepancies in prostate cancer detection by the physical examination were sufficient to account for the observed differences between subgroups.

Pavuk et al. [72] undertook an analysis of serum TCDD levels measured in 1987 (mostly), 1992, or 1997 and cancer risk among the comparison subjects only, and found no significant association between serum TCDD quartile and prostate cancer incidence, with no apparent dose–response trend (*P* = 0.72) (Table 1; Figure S1). However, risk of prostate cancer increased with longer duration of military service in Southeast Asia, with significant excesses among those who served for 2.1–3.7 years (RR = 2.2 [1.0–4.9]; 28 cases) or 3.7–16.4 years (RR = 2.4 [1.1–5.2]; 36 cases) versus 0.8–1.3 years (8 cases; *P*_trend_ = 0.003). These findings suggest that exposures in Southeast Asia other than Agent Orange, or factors associated with longer duration of military service, may be responsible for observed associations with prostate cancer. The correlation between serum TCDD levels and time served in Southeast Asia was not reported, but it appeared to be modest, based on a table showing quartiles of serum TCDD by quartiles of years in Southeast Asia.

Finally, in the most recent analysis of prostate cancer incidence in the Air Force Health Study, estimated 20-year cumulative TCDD level was not significantly associated with prostate cancer risk [73]. The adjusted RR for low cumulative TCDD in Ranch Hands (dichotomized at the median of 443 ppt-years) versus comparison subjects was 1.02 (0.67–1.55; 31 cases), and that for high cumulative TCDD in Ranch Hands was 1.22 (0.79–1.89; 28 cases) (*P*_trend_ = 0.42) (Table 1; Figure S1). A positive association was detected among those who completed their last tour of duty in Southeast Asia before 1969 (RR for high cumulative TCDD = 2.27 [1.11–4.66]; 15 cases; *P*_trend_ = 0.04) and among those who served for less than two years (RR for high cumulative TCDD = 2.15 [1.03–4.48]; 14 cases; *P*_trend_ = 0.03). When Ranch Hands and comparison subjects were analyzed separately, high versus low cumulative TCDD was not significantly associated with prostate cancer risk in either group. Time served in Southeast Asia and last year of duty also were not significantly associated with prostate cancer risk among Ranch Hands, but longer time served in Southeast Asia (dichotomized at 789 days) was significantly associated with increased risk among comparison subjects (RR = 2.18 [1.27–3.76]; 63 cases). Of note, the distribution of Gleason score (disease grade indicating likelihood of tumor spread) did not differ significantly between the 62 Ranch Hand prostate cancer cases and the 89 comparison subject cases (*P* = 0.44). This finding suggests that the two groups were equally affected by the detection of low-grade disease by means of PSA testing.

Taken together, results from the Air Force Health Study demonstrate no clear association between TCDD exposure and prostate cancer risk. The higher risk in both Ranch Hands and comparison subjects relative to the U.S. general population, combined with the lack of a difference between Ranch Hands and comparison subjects, as well as the absence of a significant difference in prostate cancer mortality between study subjects and the general population, suggests that increased prostate cancer screening among study participants compared with the general population may be responsible for much or all of the observed excess incidence. The positive associations within certain subgroups selected based on shorter duration of military service or geographic location exclusively in or outside of Vietnam were not well justified in advance, and were potentially biased or due to chance if numerous subgroups were tested.

Methodological strengths and limitations of the Air Force Health Study have been thoroughly discussed (e.g., [23, 32, 76–81]). Overall, exposure assessment is a major strength of this study. The Air Force Health Study is the only study of Vietnam veterans to have obtained serum TCDD measurements for all subjects included in dose–response analyses, thereby enabling identification of the most highly-exposed subjects. These measurements demonstrated that Ranch Hands were sufficiently exposed to TCDD that serum levels were detectable more than 15 years after their last service in Vietnam. Moreover, their TCDD levels exceeded those among U.S. Army Chemical Corps veterans (discussed below) and U.S. Army ground combat troops who served in heavily sprayed areas of Vietnam (among whom serum TCDD levels were indistinguishable from troops with no service in Vietnam or even the general public [82]). Although the estimates of past TCDD exposure could have been based on some incorrect assumptions, the substantial difference in average serum TCDD concentrations between Ranch Hands and comparison subjects indicates that the impact on estimated associations was probably modest. Thus, the persistently elevated serum TCDD levels in the majority of Ranch Hands make this group the primary bellwether for the health effects of Agent Orange/TCDD.

Another key strength of this study is the inclusion of a comparison group of veterans matched on age, race, rank, and military occupation and controlling for military service involving aircraft missions in Southeast Asia, thereby making the groups equivalent in terms of “combat-induced physiologic, psychophysiologic, and other related morbidity and mortality disorders,” as well as “the effects of alcohol consumption, the use of chemoprophylactic and/or illicit drugs, and the acquisition of tropical diseases associated with life in [Southeast Asia]” [83]. Notably, this strength was nullified in external comparisons of Ranch Hands with the general population, as conducted by Akhtar et al. [63]. With the exception of those analyses, given the unique advantages of the Air Force Health Study, its results provide compelling evidence against a strong causal relationship between Agent Orange/TCDD and prostate cancer, although the number of cases may be insufficient to detect statistically significant differences in the range of RRs and SIRs reported.

#### Army Chemical Corps study

Members of 22 U.S. Army Chemical Corps units assigned to South Vietnam between 1966 and 1971 were responsible for the storage, handling, mixing, application, and equipment maintenance of tear gas, burning agents, and herbicides, including Agent Orange [84]. Due to these job responsibilities, Army Chemical Corps personnel in Vietnam were assumed to have had, on average, much higher levels of Agent Orange exposure than other veterans (excluding Ranch Hands). Among Army Chemical Corps veterans, serum TCDD levels were highest in those who were stationed in Vietnam and reported having sprayed herbicides (mean = 4.3 ppt lipid-corrected, range 0.5–85.8), followed by herbicide sprayers not stationed in Vietnam (3.1, 0.8–9.6), non-sprayers stationed in Vietnam (2.70, 0.6–27.7), and non-sprayers not stationed in Vietnam (2.1, 0.4–12.5) [85]. Levels of six other dioxin congeners did not differ significantly between Vietnam sprayers and non-Vietnam non-sprayers [86]. In an initial cohort of 894 Army Chemical Corps members assigned to Vietnam, no prostate cancer deaths had occurred as of the end of 1987 (expected number of deaths not stated, but probably near zero), and no incident prostate cancers were identified from the U.S. Department of Veterans Affairs (VA) Agent Orange Registry (which collects information on veterans who claim Agent Orange exposure and undergo a special physical examination at a VA medical center) as of 1988 or VA inpatient discharges as of 1986 [83].

Subsequently the cohort was expanded to include 2,872 male U.S. Army veterans with at least one assignment to the Chemical Corps in Vietnam, and a comparison group of 2,737 male U.S. Army veterans last discharged from the Chemical Corps but never assigned to Southeast Asia [87]. Through 2005, five deaths from prostate cancer had occurred in Vietnam Chemical Corps members versus two in comparison subjects, resulting in an adjusted RR of 1.02 (0.19–5.64) (Table 1; Figure S1). Compared with the general U.S. population, the SMR for prostate cancer was 1.05 (0.34–2.45) among Chemical Corps members who served in Vietnam and 0.95 (0.12–3.43) among those who served outside Southeast Asia.

Despite its small size and lack of quantitative exposure estimates in Vietnam Chemical Corps members, this study is strengthened by its focus on Vietnam veterans with known military exposure to herbicides, its long and highly complete follow-up, and its use of a comparable group of veterans without military service in Southeast Asia (and, consequently, little chance of exposure to Agent Orange). Due to high survival rates, prostate cancer mortality is not a close proxy for incidence, but associations with prostate cancer mortality are less susceptible to diagnostic bias. Overall, these findings suggest little to no excess of prostate cancer mortality in Army Chemical Corps veterans who served in Vietnam, relative to their peers and U.S. males in general.

#### Other studies

Other epidemiological studies of prostate cancer have relied in part on self-reported exposure to Agent Orange, which has been shown not to be a valid indicator of serum TCDD levels [81]. Many of these studies also used the VA’s presumptive definition of Agent Orange exposure, which classifies veterans as exposed if they served for any length of time in Vietnam (including brief visits ashore and service on inland waterways) between January 9, 1962, and May 7, 1975, or in or near the Korean demilitarized zone anytime between April 1, 1968, and August 31, 1971 [88]. This definition of exposure is inconsistent with considerable evidence that ground troops in Vietnam were not appreciably exposed to Agent Orange [89]. Therefore, a major concern in such studies is exposure misclassification, which may differ between prostate cancer cases and non-cases if cases are more likely to report past exposure or to undergo a VA Agent Orange Registry health exam, or if veterans designated as exposed are more likely to undergo prostate cancer screening and/or diagnostic work-up. Importantly, such misclassification should not be assumed to be non-differential, and even exactly non-differential misclassification can often lead to overestimated RRs [90, 91]. Overall, given the unreliability of self-reported and geography-based Agent Orange exposure, the results of these studies cannot be interpreted as valid estimates of the association between Agent Orange/TCDD exposure and prostate cancer risk.

Of 400 consecutive veterans referred for prostate needle biopsy at the Palo Alto, California, VA medical center in 1998–2000, 32 (8 %) self-reported on a standard registration questionnaire that they had been exposed to Agent Orange [5]. The 32 exposed patients were age-matched to a comparison group of 96 unexposed subjects selected from the remaining 368 veterans who did not report Agent Orange exposure, and prostate cancer incidence and related characteristics were compared between the groups. Military service in Vietnam was not required for eligibility. No significant difference was detected in the proportion of exposed patients (40.4 %) versus unexposed patients (34.6 %) who were subsequently diagnosed with prostate cancer (*P* = 0.15), nor did the proportion of cases with Gleason score ≥7 differ significantly by exposure status (Table 1). Mean PSA levels, overall frequency of referral for prostate biopsy, and mean length of cancer in biopsy also did not vary between the exposed and unexposed groups, whereas exposed patients were, on average, 5 years younger at referral for prostate biopsy than the overall group of 400 referred patients (*P* = 0.01), suggesting potential differences in diagnostic intensity by exposure status. On the other hand, authors reported that an average of 1.07 % of exposed patients at their facility were referred annually for prostate biopsy, compared with 1.33 % of unexposed patients, potentially resulting in modest underestimation of the association due to diagnostic bias. Probable exposure misclassification could have biased estimates in either direction, and the number of subjects was modest. Overall, this study provides no support for an increased risk of overall or high-grade prostate cancer in veterans who self-report exposure to Agent Orange.

In a VA hospital-based case–control study set in Ann Arbor, Michigan, investigators compared self-reported Agent Orange exposure, as recorded in the computerized medical record, between 47 prostate cancer cases diagnosed in 2000–2001 and born between 1935 and 1953 (to “encompass men who were the appropriate age for potential military service in Vietnam”) and 142 age-matched controls selected randomly from the hospital’s general medicine clinic [92]. The proportion of cases (29.8 %) and controls (34.5 %) who reported military service in Vietnam did not differ significantly. However, the proportion who reported Agent Orange exposure was non-significantly higher among cases (23.4 %) than controls (12.0 %) (adjusted OR = 2.06 [0.81–5.23]) (Table 1; Figure S1). The proportion of non-Vietnam veterans who reported Agent Orange exposure was not stated. Mean age at diagnosis, Gleason score, and pathologic stage did not differ significantly between prostate cancer cases with and without Agent Orange exposure. Whether Agent Orange exposure was self-reported before or after prostate cancer diagnosis was unknown, raising the possibility of differential exposure misclassification due to recall bias. The authors also raised the possibility of selection bias due to exclusion of subjects with missing data, and noted the small number of subjects. Thus, this methodologically weak study offers no reliable evidence of any association between self-reported Agent Orange exposure and prostate cancer risk in veterans.

Another VA study based in northern California evaluated prostate cancer incidence ascertained from electronic medical records between 1998 and 2006 among 6,214 Vietnam veterans who were classified as exposed to Agent Orange (239 cases) and 6,930 Vietnam veterans classified as unexposed (124 cases) [34]. Exposure status was based on self-reported Agent Orange exposure on the initial application for medical benefits and having been stationed in “known areas that were sprayed with Agent Orange during 1962 through 1971” (i.e., 27–36 years prior to case ascertainment). The definition of “known areas” was not further elaborated, making it unclear whether, for example, the entire country of Vietnam was considered to be an area sprayed with Agent Orange. Seven veterans who initially claimed no exposure to Agent Orange but changed their self-reported exposure status after developing prostate cancer were excluded, whereas 38 patients who first reported Agent Orange exposure after prostate cancer diagnosis were retained in the analysis; the remaining 231 cases who reported Agent Orange exposure apparently did so before prostate cancer diagnosis. The timing of exposure ascertainment relative to prostate cancer diagnosis or study entry was unclear for the remaining study subjects.

The frequency of PSA screening, the proportion of patients evaluated by a urologist for elevated PSA, and the proportion who subsequently underwent biopsy did not differ significantly by exposure status (Table 1) [34]. Risk of prostate cancer was significantly higher among exposed than unexposed veterans (adjusted OR = 4.83 [3.42–6.81]) (Figure S1). Adjusted ORs were also significantly elevated for high-grade prostate cancer (OR = 2.59 [1.30–5.13]) and metastatic prostate cancer at diagnosis (OR = 4.32 [1.34–13.96]). After excluding the 38 patients who reported Agent Orange exposure after prostate cancer diagnosis, the association was substantially attenuated but still statistically significant (OR = 1.85 [1.47–2.31]). Among the prostate cancer cases, those classified as exposed to Agent Orange, compared with those classified as unexposed, were significantly younger at diagnosis, had a higher mean Gleason score, had a higher proportion of high-grade disease, were more likely to present with metastasis, and were less likely to have a family history of prostate cancer, but did not differ significantly in clinical stage or PSA level. It is somewhat surprising that the prevalence of PSA testing and early-stage prostate cancer did not differ by exposure status, given that veterans with self-reported exposure were invited to undergo PSA testing and digital rectal examination; therefore, one might expect that exposed veterans would be more likely to undergo these procedures than the non-exposed. As expressed by Air Force Health Study investigators [93], the “serious misclassification of Agent Orange/TCDD exposure” in the study by Chamie et al. [34] “undermines the validity of their conclusions.” Moreover, the implausibly large estimated RRs suggest a chance finding, potential selection bias, differential exposure misclassification, or other sources of bias, although possible bias was difficult to evaluate due to the ambiguous description of study methods.

Among 2,720 veterans referred to the Portland, Oregon, VA Medical Center for initial prostate biopsy and without a history of prostate cancer, 203 were classified as exposed to Agent Orange based on self-report at the time of hospital enrollment (before prostate biopsy) or military service in “a location where [Agent Orange] was known to have been used,” and the remaining 2,517 were classified as unexposed [33]. Subjects were not restricted to Vietnam or Vietnam-era veterans. Prostate cancer was diagnosed in 74 (36 %) exposed veterans, including 40 (20 %) with Gleason score ≥7, and in 822 (33 %) unexposed veterans, including 419 (17 %) with Gleason score ≥7.

Despite these relatively small differences by exposure status, the adjusted OR for prostate cancer (vs. no prostate cancer) in exposed versus unexposed veterans was 1.52 (1.07–2.13) (Table 1; Figure S1) [33]. Comparing high-grade (Gleason score ≥7) disease with none, the adjusted OR was 1.75 (1.12–2.74), whereas that for low-grade disease was 1.24 (0.81–1.91). However, military service branch was not significantly associated with prostate cancer risk among veterans classified as exposed to Agent Orange (results not shown by authors). Veterans with reported Agent Orange exposure were significantly younger at biopsy than unexposed veterans, and age at diagnosis was also younger and mean PSA (but not PSA density) was lower in exposed than unexposed cases, suggesting greater diagnostic intensity among exposed subjects. Yet, the stronger association of Agent Orange exposure with high-grade than low-grade disease runs counter to the anticipated effect of diagnostic bias. The high potential for exposure misclassification based on self-report and the VA’s presumptive definition of Agent Orange exposure renders the results difficult to interpret. Thus, this study does not offer reliable evidence of a significant positive association between actual exposure to Agent Orange and risk of high-grade prostate cancer.

A cross-sectional postal survey of 114,562 Korean veterans of the Vietnam War (70 % of 164,208 contacted) was conducted in 2004 to evaluate associations between Agent Orange exposure and self-reported disease outcomes [94]. Agent Orange exposure was assessed based on self-report (categorized as no, low, moderate, or high) and based on the proximity of the military unit (battalion or company based on self-reported survey data; division or brigade based on Ministry of Defense records) to sprayed areas, using the exposure opportunity index model E4 developed by Stellman et al. [95].

Self-perceived exposure to Agent Orange was associated with an increased prevalence of prostate cancer (OR for high vs. no exposure = 1.91 [1.69–2.16]) (Table 1; Figure S1) [94]. However, modeled exposure to Agent Orange was not significantly associated with prostate cancer, either at the division/brigade level or at the more detailed battalion/company level (OR for low exposure vs. none = 1.04 [0.92–1.17]; moderate exposure vs. none = 1.04 [0.91–1.19]; high exposure vs. none = 1.11 [0.97–1.27]; *P*_trend_ = 0.15). The combination of self-reported exposure and self-reported disease is highly susceptible to information bias. The validity and reliability of the exposure opportunity index model have also been questioned on the basis of widely heterogeneous exposure estimates [96]. Moreover, serum TCDD levels in 102 Korean Vietnam veterans in 2007 were poorly correlated with either perceived self-reported exposure (r^2^ = 0.129) or proximity-based exposure (r^2^ = 0.073 with division/brigade-level exposure; r^2^ = 0.159 with battalion/company-level exposure), nor with military unit (*P* = 0.67 for support vs. combat unit as a predictor of serum TCDD concentration) [97], and average TCDD levels (mean = 1.2 ppt, median = 0.9 ppt) were lower than in U.S. Vietnam-era veterans stationed in the continental U.S. or Germany (mean = 4.1 ppt) [81]. Thus, the interpretation of perceived and modeled Agent Orange exposure in this study is unclear, and the results do not support an association between actual Agent Orange exposure and prostate cancer in Korean Vietnam veterans.

Three other studies of prostate cancer in veterans compared patient and disease characteristics between prostate cancer patients with and without reported Agent Orange exposure (Table 1) [98–100]. These studies do not address the association between Agent Orange exposure and prostate cancer risk. However, they can provide information on differences in diagnostic intensity between exposed and unexposed veterans (although not between veterans and the general population), and address the hypothesis raised by Ansbaugh et al. [33] and Chamie et al. [34] that Agent Orange exposure is associated with higher-grade disease.

At a West Virginia cancer center between 1995 and 2005, 29 Vietnam veterans with Agent Orange exposure determined by eligibility for VA benefits and 52 Vietnam veterans without Agent Orange exposure underwent permanent brachytherapy for prostate cancer [100]. Those with Agent Orange exposure were significantly younger at the time of implantation and had significantly higher pre-treatment PSA, but did not differ significantly in terms of Gleason score, percent positive biopsies, prostate volume, clinical stage, perineural invasion, or risk group (based on a combination of PSA, Gleason score, and stage), indicating no significant difference in diagnostic intensity or disease characteristics by VA-designated Agent Orange status.

Among 1,495 veterans at four VA medical centers who underwent radical prostatectomy for prostate cancer in 1988–2007, including 206 classified in the electronic medical record as having been exposed to Agent Orange and 1,289 classified as unexposed, exposed patients were significantly more likely to be younger at the time of radical prostatectomy, have lower-stage disease, and have a lower preoperative PSA level [99]. However, no significant group differences were observed in the distribution of biopsy or pathological Gleason score, the prevalence of lymph node metastasis, or the odds of having high-grade disease, positive surgical margins, or seminal vesicle invasion. These results may suggest heightened surveillance for prostate cancer among veterans reporting Agent Orange exposure, but no significant difference in Gleason score or other disease characteristics.

Among 93 veterans at the Augusta, Georgia, VA medical center who underwent radical prostatectomy for prostate cancer in 2005–2009, those classified in the electronic medical records (based on self-report and military records confirming military service “in an area in which [Agent Orange] had been sprayed”) as exposed were older at the time of radical prostatectomy than those classified as unexposed, and were less likely to have positive surgical margins, but did not differ significantly in terms of preoperative PSA level, prostate volume, biopsy or prostatectomy Gleason score, tumor pathological stage, extracapsular extension, seminal vesicle invasion, lymph node metastasis, or subsequent mortality (with a median follow-up of 64 months) [98]. Results were similar when patients were classified as having high or low dioxin-like toxic equivalency values measured in subcutaneous adipose tissue. Although the findings regarding age at prostatectomy, preoperative PSA level, and tumor stage differ from those of Shah et al. [99], with less evidence of disparate diagnostic intensity, they again suggest no significant relationship between self-reported/geography-based Agent Orange exposure and clinical disease characteristics.

In summary, three VA hospital-based studies in the U.S. [33, 34, 92] and a cross-sectional study of prostate cancer prevalence in Korea [94] reported a significant positive association between self-reported or geography-based Agent Orange exposure and prostate cancer, whereas one VA-based study did not [5]. Also, two studies found a significant positive association between self-reported or geography-based Agent Orange exposure and risk of high-grade prostate cancer [33, 34], whereas five did not [5, 92, 98–100]. Only the Korean investigators attempted to validate self-reported Agent Orange exposure by comparison with serum TCDD levels, and they found no correlation between the two [97]. The same lack of association between serum TCDD levels and self-reported or proximity-based estimated Agent Orange exposure has been demonstrated in U.S. veterans [81]. In light of the high probability of severe exposure misclassification, these studies cannot reliably be interpreted as unbiased analyses of associations with actual Agent Orange exposure. Combined with concerns about potential recall bias, selection/diagnostic bias, and other methodological problems, the unknown interpretation of self-reported Agent Orange exposure renders these studies essentially uninformative with regard to the question of a causal effect of Agent Orange or TCDD on prostate cancer.

### Studies of Vietnam veterans without estimated Agent Orange/TCDD exposure

In its Veterans and Agent Orange updates, the IOM Committee takes into consideration studies of Vietnam veterans that lack estimates of exposure to Agent Orange/TCDD or restriction to a subgroup of veterans known to have had high potential for Agent Orange exposure [23–32]. As stated in the most recent (2012) update, Vietnam veterans “are assumed to have had a higher probability of exposure to the [chemicals of interest] than people who did not serve in Vietnam, whether or not their individual exposures are characterized beyond the mere fact that they were deployed to Vietnam” [23]. However, comparisons of all Vietnam veterans with veterans who served outside of Southeast Asia must be interpreted with caution due to the high potential for misclassification of Agent Orange exposure, as well as possible differences in diagnostic intensity. As noted by the U.S. Centers for Disease Control and Prevention (CDC), differences between Vietnam and non-Vietnam veterans could include “psychological stresses of war, possible exposure to various infectious diseases prevalent in Vietnam, possible misuse of drugs and alcohol, and possible exposure to the defoliant Agent Orange, as well as many unknown exposures” [101]. Such exposures might contribute to increased health care usage and, hence, diagnostic intensity among Vietnam veterans [102–104]. Comparisons with general populations are especially susceptible to this type of bias due to the generally greater health care access and/or usage among veterans than non-veterans [68, 105]. Thus, associations with military service in Vietnam cannot reliably be attributed to exposure to Agent Orange/TCDD, and such studies are only briefly discussed here because they are not informative about causal associations between Agent Orange/TCDD exposure and prostate cancer risk.

The Government of Australia conducted a series of retrospective cohort studies comparing Australian veterans of the Vietnam War with either conscripts who served only in Australia or the general population of Australia. Australian military personnel in Vietnam were mostly stationed in Phuoc Tuy Province, which was not heavily sprayed with Agent Orange; thus, the average exposure of Australian troops to Agent Orange was probably low, if any [106]. Analyses that compared approximately 19,000 Australian male National Service conscripts who served in Vietnam with approximately 25,000 who served only in Australia found no significant difference in genitourinary cancer mortality (RR = 0.7 [0.2–3]) [107], prostate cancer incidence (RR = 1.05 [0.75–1.48]) [109], or prostate cancer mortality (RR = 0.00 [0.00–1.41]; 0 vs. 5 deaths) between the groups (Table 2; Figure S2).

Other epidemiologic studies of Australian veterans of the Vietnam War were based on comparisons between Vietnam veterans and the general Australian population, rather than veterans stationed elsewhere. Although most (but not all [109]) of these analyses found significant elevations in prostate cancer mortality [110, 112], prevalence [114, 115], and incidence [116] among Australian Vietnam veterans (Table 2; Figure S2), the excess cannot reliably be attributed to any specific difference, including Agent Orange or any other exposure, between veterans and non-veterans.

The U.S. CDC designed the Vietnam Experience Study to evaluate the health consequences of military service in Vietnam, where “Vietnam Experience” was used as “a generic term for a wide range of health-influencing exposures operating among those who served in the military in Vietnam” [101]. The authors noted that “[t]hese factors are unmeasured in this study; therefore, it is not possible to examine directly their relation to mortality.” Postservice mortality was compared between 9,324 Vietnam veterans and 8,989 non-Vietnam veterans who served in Korea, Germany, or the U.S. during the Vietnam era. As of the end of 1983, no deaths from prostate cancer had occurred among either Vietnam or non-Vietnam veterans (Table 2) [101]. With additional follow-up through 2000, one death from prostate cancer had occurred among Vietnam veterans (mortality rate = 0.4 per 100,000 person-years), compared with three deaths among non-Vietnam veterans (1.1 per 100,000 person-years) [117].

Notably, the CDC designed another study specifically to evaluate the potential health effects of Agent Orange exposure. However, this study was stopped after the exposure assessment phase because serum TCDD levels measured in 1987 did not differ between veterans who were stationed in the most heavily sprayed area of Vietnam (III Corps) and veterans who served in Germany or the U.S. during the same years [81]. Therefore, the authors concluded that “most U.S. Army ground troops who served in Vietnam were not heavily exposed to TCDD, except perhaps men whose jobs involved handling herbicides” [81], and that investigation of the potential health effects of Agent Orange exposure in Vietnam veterans overall was infeasible.

A case–control study of 606 prostate cancer cases (61 % participation) and 471 age-matched controls randomly selected from electoral rolls (43 % participation) in Western Australia found a non-significant increase in prostate cancer risk in association between self-reported military service in Vietnam (age-adjusted OR = 2.12 [0.88–5.06]) [118]. The estimated association with Vietnam service was similar after adjusting for family history of prostate cancer or restriction to patients with Gleason score ≥7 (Table 2; Figure S2). Potential exposure to Agent Orange was not assessed, but as stated by others [106], was likely to have been low or non-existent among Australian troops in Vietnam.

A retrospective cohort study of 2,783 (out of 3,322 eligible) male New Zealand veterans who served in Vietnam found no significant increase in prostate cancer incidence (SIR = 1.17 [0.98–1.39]) or mortality (SMR = 1.03 [0.55–1.76]) in comparison with the general New Zealand population (Table 2; Figure S2) [119]. The majority of New Zealand troops served alongside the Australian Army in Phuoc Tuy Province [119], where Agent Orange spraying was limited [106].

Another retrospective cohort study of 185,265 male Korean veterans of the Vietnam War detected a significant excess of prostate cancer incidence (SIR = 1.22 [1.02–1.46]) among all Vietnam veterans combined, compared with the general male population of Korea (Table 2; Figure S2) [120]. This excess was restricted to officers (SIR = 2.49 [1.93–3.21]), whereas enlisted soldiers and non-commissioned officers had a non-significant deficit of prostate cancer incidence (SIRs = 0.85 [0.64–1.14] and 0.81 [0.51–1.29], respectively). Given that serum TCDD levels did not vary by military rank [97], the prostate cancer excess in officers cannot reliably be attributed to differences in Agent Orange exposure.

Proportionate mortality studies, which compare the percentage of all deaths from a specified cause between an exposed group of interest and an unexposed or general population group, can give rise to misleading conclusions about risk associations if they are used to compare groups with different distributions of causes of death unrelated to the exposure under study. Therefore, they do not provide useful information about the association between Agent Orange/TCDD exposure and prostate cancer risk, and they are listed here only for the sake of completeness. No published proportionate mortality or morbidity studies of U.S. Vietnam versus non-Vietnam veterans, including those in New York State [121], Wisconsin [122], West Virginia [123], Michigan [124], Massachusetts [125, 126], and the U.S. Army and Marine Corps [127, 128], found a significant excess of prostate cancer incidence or mortality among Vietnam veterans (Table 2; Figure S2).

In summary, studies in Australia, New Zealand, and Korea found that prostate cancer incidence, mortality, and/or prevalence were higher in Vietnam veterans than in the general population [110–116, 118–120], but comparisons between Vietnam and non-Vietnam veterans in Australia [107, 109] and the U.S. [101, 117, 121, 122, 124–128] found no such excess. Thus, the collective findings suggest that military service specifically in Vietnam does not confer an increased risk of prostate cancer beyond that associated with military service in general. These studies are limited by a lack of detailed exposure assessment, poor control for confounding by medical surveillance and other factors, possible selection bias, and a low probability of direct or indirect exposure to Agent Orange. Therefore, these studies—most of which found no significant association between Vietnam military service and prostate cancer—do not provide informative results regarding the potential causal effect of Agent Orange/TCDD on prostate cancer.

### Studies of manufacturers and sprayers of herbicides potentially contaminated with TCDD

The most informative industry-based studies of occupational exposure to TCDD concern two groups of workers: chemical workers involved in the production of potentially contaminated herbicides (2,4,5-T, trichlorophenol, and pentachlorophenol), and sprayers of the same herbicides (mainly 2,4,5-T). The review of the results on prostate cancer risk among herbicide manufacturers and sprayers is complicated by the fact that several partially overlapping study groups have been included in various reports. Several studies conducted in Europe and Oceania (most of which had been reported separately) were combined in a pooled analysis coordinated by IARC [129]; similarly, several US-based studies (also reported separately) were combined in a pooled analysis coordinated by the National Institute for Occupational Safety and Health (NIOSH) [135]. The results of the two pooled analyses were then reported in a combined publication, which comprised four additional German cohorts that also had been reported separately [17]. An update of the NIOSH pooled study was subsequently reported [136], as well as updated analyses of some of the individual plants. Similarly, updated analyses were reported for several of the studies included in the IARC pooled study and one of the German cohorts.

In our review, we considered the combined publication of the two pooled analyses [17], the NIOSH study update [136], as well as the subsequent individual cohort studies that provided more extensive results in terms of the size of the study group or the duration of follow-up [137–144]. One additional study of herbicide production workers was not included in any pooled analysis and is reviewed separately [145]. The relevant studies are summarized in Table 3, and are described relatively briefly in this section because of their uniformly statistically null results with respect to prostate cancer. Although false-negative findings are a possibility, whether due to insufficient statistical power, exposure misclassification, confounding, other biases, or chance, the consistency of null findings suggests that the absence of association is real.

The combined IARC/NIOSH pooled analysis comprised 21,863 male and female workers exposed to phenoxy herbicides or chlorophenols from 32 cohorts of manufacturing workers from Austria, Denmark, Finland, Germany, Italy, the Netherlands, New Zealand, Sweden, the United Kingdom, and the United States, and four cohorts of sprayers from Australia, Canada, New Zealand, and the United Kingdom (Supplemental Table S1) [17]. The period of employment and mortality follow-up varied among countries; overall, they extended from 1939 to 1992. Exposure to TCDD was defined as employment during manufacture or spraying of 2,4,5-T, pentachlorophenol, or other potentially contaminated compounds, or employment in plants with documented exposure through biomonitoring (data available for 573 workers from 10 cohorts) or environmental measurements. Published mean serum TCDD levels in subsets of workers included in the IARC/NIOSH pooled analysis and controls are shown in Table 6; these data demonstrate that phenoxy herbicide and chlorophenol production workers and sprayers experienced higher TCDD exposure than the population at large.

**Table 6:** Mean serum TCDD levels in groups of workers included in the IARC/NIOSH pooled analysis [17] and controls

Country	Exposed workers		Unexposed controls		References
	N, type	Mean (ppt)	N, type	Mean (ppt)	
Austria	9 PC	389	17 O	~14	Neuberger et al. (130)
Germany	20 P	141^a^	–	–	Flesch-Janys et al. [131]
Germany	20 PC	402^a^	–	–	Manz et al. [132]
New Zealand	9 Sp	53	9 O	6	Smith et al. [133]
Sweden	5 P	17	5 O	2	Littorin et al. [134]
United States	253 P	233	79 S	2	Fingerhut et al. [135]

P, production workers; C, chloracne patients; Sp, sprayers; S, workers from same plants as exposed workers; O, workers from other plants than exposed workers

^a^Modeled TCDD level at the end of employment

Overall, 13,831 workers from 28 cohorts (63.3 %) were classified as exposed to TCDD (73 % manufacturing workers and 27 % sprayers; 63 % from the IARC study and 37 % from the NIOSH study), and 7,553 workers (34.5 %) were classified as unexposed to TCDD (479 workers from a UK plant [2.2 %] could not be classified as to TCDD exposure). The SMR for prostate cancer was 1.11 (0.81–1.50; 43 deaths) among exposed workers and 1.10 (0.71–1.62; 25 deaths) among unexposed workers (Table 3; Figure S3). Prostate cancer results by duration of employment, time since first employment, or other indirect indicators of exposure were not reported. Similarly, separate results on herbicide manufacturers and sprayers were not reported for prostate cancer. The original IARC pooled analysis comprised 13,482 workers from 20 cohorts of production workers and sprayers exposed to phenoxy herbicides and chlorophenols [129]. A total of 30 deaths from prostate cancer were observed (SMR = 1.11 [0.75–1.58]); no results were reported according to TCDD exposure. In the original NIOSH pooled analysis, comprising 5,172 production workers, the SMR for prostate cancer was 1.22 (0.71–1.95; 17 deaths) in the whole cohort and 1.52 (0.70–2.90; 9 deaths) in the group of 1,520 workers with one or more years of employment [135].

A subsequent analysis of the NIOSH pooled study [136] provided results for mortality up to 1993, as compared with 1987 in the combined IARC/NIOSH analysis. This analysis comprised 5,132 workers employed in 12 plants, all of whom were considered exposed to TCDD. The SMR for prostate cancer, based on 28 deaths, was 1.17 (0.78–1.68) (Table 3; Figure S3).

Collins et al. [137, 138] reported two analyses of workers employed in one of the plants included in the NIOSH pooled analysis. The first analysis [137] included 773 male pentachlorophenol manufacture workers exposed to TCDD. The period of follow-up was not specified. Serum TCDD levels were available for 128 workers and were used to model TCDD exposure for the whole study cohort. The SMR for prostate cancer in the whole cohort was 1.0 (0.4–1.9), based on eight deaths (Table 3; Figure S3). When workers were categorized in tertiles according to estimated TCDD exposure, SMRs were 0.8 (0.1–2.9) for low exposure (<0.7 ppt-years; 2 deaths), 0.6 (0.1–2.4) for medium exposure (0.7–3.99 ppt-years; 2 deaths), and 1.4 (0.4–3.6) for high exposure (≥4.0 ppt-years; 4 deaths). The estimated RR of prostate cancer death per ppt-year was 1.002 (0.962–1.045). The second analysis [138] included 1,615 trichlorophenol workers with potential TCDD exposure, including 196 pentachlorophenol workers who presumably were also included in the first analysis. As in the first analysis, TCDD exposure was modeled based on serum levels, which were available for 280 workers. After excluding pentachlorophenol workers, there were 20 deaths from prostate cancer through 2003 (SMR = 1.5 [0.9–2.4]). The RR of prostate cancer death per ppb-year increase in cumulative estimated TCDD level was 1.013 (0.989–1.038; *P* = 0.29).

A further analysis included 2,122 workers at four pentachlorophenol production plants that were part of the NIOSH pooled study [139]. A subcohort of 720 workers involved in trichlorophenol production was considered exposed to TCDD. In a follow-up through 2005, eight deaths from prostate cancer were observed among the exposed workers (SMR = 1.03 [0.45–2.04]), whereas 18 prostate cancer deaths were observed among the unexposed (SMR = 0.98 [0.58–1.55]) (Table 3; Figure S3).

‘t Mannetje et al. [140] extended to 2000 the follow-up of two cohorts from New Zealand included in the IARC pooled analysis (1,025 production workers and 703 sprayers). The SMR for prostate cancer was 0.37 (0.01–2.08; 1 death) among production workers and 0.60 (0.07–2.16; 2 deaths) among sprayers (Table 3; Figure S3). The cohort study of manufacturing workers was expanded by McBride et al. [141, 142]. A first report [141] included 1,754 workers employed in the manufacturing plant between 1969 and 2003, with mortality follow-up through 2004. A total of three deaths from prostate cancer were observed (SMR = 0.54 [0.11–1.56]). All three deaths occurred among workers with less than three months of employment (SMR in this group = 0.62 [0.13–1.82]; 0.8 expected prostate cancer deaths among workers with longer duration of employment). The second analysis [142] was restricted to 1,599 workers employed between 1969 and 1988, when 2,4,5-T was used and exposure to TCDD was possible, with mortality follow-up through 2004. A total of 1,134 workers were classified as exposed to TCDD, and 465 workers were considered unexposed. Cumulative TCDD levels were estimated based on serum levels available for 346 workers. Three deaths from prostate cancer were observed in this cohort (presumably the same deaths reported in [141]); one death was among exposed workers (SMR = 0.2 [0.0–1.2]) and two deaths were among unexposed workers (SMR = 1.9 [0.2–6.7]). Results according to estimated cumulative TCDD exposure were not reported for prostate cancer.

The mortality of 2,106 male production workers employed in two Dutch plants included in the IARC pooled study was updated to 2006 [143]. A total of 539 workers in factory A and 411 workers in factory B were classified as exposed to TCDD based on either their history of employment in selected departments (or having entered them on a regular basis) or exposure to an accident that occurred in 1963 in factory A. The remaining 482 workers in factory A and 626 workers in factory B were classified as unexposed. The RR of prostate cancer for TCDD exposure was 2.93 (0.61–14.1) in factory A (based on six deaths among the exposed and two deaths among the unexposed) and 2.68 (0.48–14.9) in factory B (based on four deaths among the exposed and two deaths among the unexposed) (Table 3; Figure S3). Four out of the six exposed cases in factory A were present during the 1963 accident. Boers and colleagues [146] extended this analysis by including estimated TCDD exposure based on plasma measurements available for 187 workers. The RR of prostate cancer per log unit of TCDD (with a 10-year lag) was 1.08 (0.79–1.49). In this analysis, all workers in factory B were considered unexposed to TCDD, and when the analysis was restricted to factory A, the RR per lagged log unit of TCDD level was 1.29 (0.85, 1.94).

The mortality follow-up of one of the plants from Germany included in the IARC pooled study was extended to 2007 [144]. The cohort included workers ever employed between 1952 and 1984, when the plant was closed. Causes of death of deceased cohort members were coded by a study pathologist; expected deaths were calculated based on regional rates. Among 1,191 male workers, a total of 19 deaths from prostate cancer were observed (SMR = 1.37 [0.82–2.13]) (Table 3; Figure S3). Cumulative TCDD exposure was estimated on the basis of serum levels on a subset of workers, but results on prostate cancer were not reported according to estimated TCDD level.

The only group of workers not included in the IARC or the NIOSH pooled analysis is a cohort of 243 male workers who were involved between 1953 and 1969 in clean-up, repair, maintenance, or demolition activities at a German plant where an accident entailing release of TCDD occurred in 1953 [145]. Serum TCDD level was available for all cohort members. Follow-up for mortality and cancer incidence covered the period 1953–1992. No deaths from prostate cancer occurred in the cohort (SMR = 0 [0–1.9]), while four incident cases were observed (SIR = 1.1 [0.3–2.8]) (Table 3; Figure S3). When workers were categorized according to serum TCDD level, the SIR was 2.5 (0.5–7.4; 3 cases) in the low-exposure category (<0.1 µg/kg body weight), 1.1 (0.0–5.9; 1 case) in the medium-exposure category (0.1–0.99 µg/kg body weight), and 0.0 (0.0–2.5; 0 cases) the high-exposure category (≥1 µg/kg body weight).

Subsets of workers who developed chloracne, indicating high TCDD exposure, were identified in the NIOSH pooled cohort (630 workers) [136] and in the German accident cohort (113 workers) [145]. However, no results on risk of prostate cancer were reported for these groups of workers.

Two other studies provided limited information on prostate cancer risk associated with potential occupational exposure to TCDD-contaminated herbicides. In the Agricultural Health Study, a cohort of 55,332 male pesticide applicators from Iowa and North Carolina, no association was found between self-reported use of 2,4,5-T (or 2,4-D) and risk of prostate cancer, based on 566 cases after a mean follow-up period of 4.3 years (detailed results not reported) [147]. In a population-based case–control study in Western Australia, self-reported exposure to phenoxy herbicides was not associated with risk of prostate cancer (OR = 1.00 [0.61–1.63]; 40 exposed cases) [148].

In summary, although a formal combination of results is complicated by the overlap among reports, studies of manufacturers and sprayers of TCDD-contaminated herbicides consistently indicate no significant increase in risk of prostate cancer. This conclusion comports with that of a meta-analysis of 20 cohort studies of phenoxy herbicide manufacturing workers published through 2003, in which the combined SMR for prostate cancer was 1.16 (0.85–1.57) [149]. Limitations of the available data include the fact that most studies reported only results for prostate cancer mortality and that the number of workers with high documented exposure is relatively small. Workers with presumably different levels of TCDD exposure were combined for analysis, resulting in some degree of exposure misclassification. Despite these limitations, a substantial proportion of manufacturers and sprayers included in these cohorts did experience considerably higher exposure to TCDD than the general population, as demonstrated by the serum TCDD levels measured in several subsets of workers. Thus, the results of these studies provide solid evidence against a significant positive association between TCDD or herbicides contaminated with TCDD, including 2,4,5-T, and prostate cancer.

### Studies of the Seveso industrial accident

Part of the population living in six Italian municipalities was exposed to TCDD as a result of an accident in a trichlorophenol production plant in Seveso in 1976. Several reports on cancer mortality or incidence in this area have been published; the most recent analyses are based on follow-up for cancer mortality through 2001 [150] and cancer incidence through 1996 [151]. The design and results of these studies are summarized in Table 4, and results are shown in Figure S3.

The exposed group was divided among those living in 1976 (or migrating during 1976–1985) in zone A, with the highest exposure (median serum TCDD level in 1976 = 447.0 ppt); zone B, with intermediate exposure (median serum TCDD = 94.0 ppt); and zone R, with low exposure (median TCDD level = 48.0 ppt). The reference group comprised the remaining areas of the six municipalities, as well as five surrounding municipalities. Cancer incidence was based on record linkage with the regional population-based cancer registry, and the mortality follow-up was based on vital status information obtained from the municipalities of residence and on cause of death obtained from the regional statistical office.

The cancer incidence analysis was based on people living in the contaminated area (352 men in area A, 2,471 men in zone B, 15,715 men in zone R, and 88,349 men in the reference zone) [151]. This analysis included zero cases of prostate cancer in zone A (expected cases not reported, but estimated as approximately 1 case, based on the rates in zones B and R), seven cases in zone B (RR = 0.94 [0.45–1.99]), and 39 cases in zone R (RR = 0.75 [0.54–1.05]) (Table 4; Figure S3).

The cancer mortality analysis included also those individuals who were born or immigrated into the study area between 1976 and 1985 (390 men in area A, 3017 men in area B, 19,199 men in area R, and 139,516 men in the reference area) [150]. The observed deaths from prostate cancer were one in zone A (RR = 0.87 [0.12–6.17]), eight in zone B (RR = 0.88 [0.44–1.77]), and 65 in zone R (RR = 1.06 [0.81–1.38]) (Table 4; Figure S3).

In summary, there is no evidence of an increased risk of prostate cancer in the community exposed to the Seveso accident with follow-up extending over 20–25 years. While the number of individuals in the zone with highest exposure is relatively small, and exposure was classified with some error based on geographic zone of residence instead of individual serum TCDD level, the results in the combined zones A and B are not compatible with a significant association between TCDD exposure and prostate cancer.

A recent meta-analysis of cohort studies of TCDD and prostate cancer calculated a meta-SMR of 1.26 (1.00–1.57) based on 13 military and occupational cohorts (one of which reported an SIR) and a meta-RR of 1.04 (0.84–1.28) based on four military and community cohorts [152]. This meta-analysis had a number of important flaws, such as the omission of several relevant articles [17, 137, 142, 144, 146, 151], the selection of results from some exposed subcohorts but not others [140], the equal weighting of RRs or SIRs for subjects with low, medium, or high TCDD exposure within a given cohort [145, 150], and the conflation of incidence and mortality data and heterogeneous military, occupational, and environmental exposure levels. Overall, this study offers no additional insight into the relationship between TCDD and prostate cancer risk. This study also demonstrates that meta-analysis may be poorly suited for synthesizing results of observational studies with heterogeneous settings, methods, and exposure levels that require careful interpretation of individual and collective results for causal inference [59, 60].

## Discussion

The best available epidemiologic studies of exposure to Agent Orange/TCDD show no significant association with prostate cancer incidence or mortality. Specifically, the only two studies of Vietnam veterans with exposure to Agent Orange confirmed by elevated serum TCDD levels—namely, the Air Force Health Study and the Army Chemical Corps study—found no significant increase in prostate cancer incidence [64, 73] or mortality [62, 84, 87] in exposed veterans overall. Likewise, occupational cohort studies of industrial exposure to TCDD-contaminated herbicides and studies of the Seveso community cohort—considered by IARC to be the most informative epidemiologic studies for evaluating the carcinogenic effects of TCDD in humans—consistently found no significant association with prostate cancer incidence or mortality [17, 136–145, 150, 151]. RR point estimates in these studies were fairly evenly distributed above, below, and at the null value of 1.0, with no evidence that subgroups with greater exposure experienced a higher risk. The Air Force Health Study main analyses, the Army Chemical Corps study, the occupational cohort studies, and the Seveso cohort study are accorded the greatest weight in evaluation of the overall epidemiologic literature on Agent Orange/TCDD exposure and prostate cancer because these are the only studies in which subjects designated as exposed were confirmed to have elevated serum levels of TCDD.

In his well-known and widely implemented guidelines for the evaluation of potential causal associations, Sir Austin Bradford Hill specified that the guidelines were intended to be applied to “an association between two variables, perfectly clear-cut and beyond what we would care to attribute to the play of chance. What aspects of that association should we especially consider before deciding that the most likely interpretation of it is causation?” [153]. In the absence of such a “clear-cut” association—indeed, in the presence of considerable evidence indicating no association, as in the case of Agent Orange/TCDD and prostate cancer—characteristics such as the strength, consistency, specificity, temporality, exposure–response gradient, plausibility, and coherence of a potential exposure-disease relationship are not strictly applicable. Nonetheless, based on the most informative epidemiologic studies, we can state that the apparent association is not strong, that results across studies are consistent in demonstrating the absence of any increase in prostate cancer risk, and that there is no positive exposure–response gradient.

The null results are also coherent with toxicological data from experimental animal studies. Specifically, although TCDD promotes a variety of tumors in different strains of rats, mice, and hamsters through a non-genotoxic mechanism believed to involve the Ah receptor [11, 12, 14–16], benign or malignant tumors of the prostate are not among those observed in excess in experimental animals. 2,4,5-T itself, in the absence of TCDD, has been found not to increase tumor incidence in experimental rodents [154]. Similarly, 2,4-D—the other ingredient of Agent Orange—demonstrates no apparent oncogenic effect in experimental animals [155–157]. Thus, animal evidence supports the results of epidemiologic studies showing no significant association between Agent Orange or TCDD exposure and prostate cancer risk.

The only studies that found a significant positive association with prostate cancer were those in which the exposure under evaluation was self-reported or geography-based Agent Orange exposure [33, 34, 92, 94] or Vietnam military service overall in comparison with the general population [63, 110–116, 118–120]; or, in the Air Force Health Study, where the analysis was restricted to veterans with a shorter duration of military service or where Ranch Hands who served exclusively in Vietnam were compared with subjects who served exclusively outside of Vietnam [63]. As discussed earlier, self-reported and proximity-based Agent Orange exposure estimates are not closely correlated with serum TCDD levels [82, 97]. Therefore, profound exposure misclassification, combined with potential selection bias, largely invalidates the findings of these studies, which cannot contribute useful information about the association between Agent Orange/TCDD exposure and prostate cancer risk. Confounding by diagnostic intensity, particularly PSA testing, is a major concern in studies that compare Vietnam veterans with the general population, as well as in the subgroup analyses of the Air Force Health Study, where chance (given probable testing of numerous subgroups) is another important potential explanation for the reported associations. Taken together with the null results of the more reliable studies identified above, the positive findings from these selected studies of veterans are more likely to be spurious than valid.

In conclusion, studies of Vietnam veterans, herbicide manufacturers or sprayers, and community members known to be highly exposed to Agent Orange or TCDD consistently demonstrate the absence of a significant increase in prostate cancer incidence or mortality in these groups. Several bias-prone studies of veterans with poorly characterized Agent Orange exposure reported an excess of prostate cancer, but such findings cannot reliably be attributed to an effect of Agent Orange/TCDD rather than confounding or bias. Toxicological findings in animals are also coherent with the absence of a carcinogenic effect of TCDD on the prostate. Overall, the scientific evidence establishes no causal association between exposure to Agent Orange or TCDD and prostate cancer in humans. However, the most rigorously designed studies may lack sufficient power to determine whether RRs of the magnitude generally reported (approximately 0.3–3.0) were statistically significantly different from 1.0. Development of more sensitive biomarkers of past exposure to Agent Orange or TCDD could enable larger studies to rule out a causal association more conclusively.

## Electronic supplementary material

Below is the link to the electronic supplementary material.

Supplementary material 1 (DOCX 176 kb)

## Abbreviations

2,4-D: 2,4-Dichlorophenoxyacetic acid
2,4,5-T: 2,4,5-Trichlorophenoxyacetic acid
Ah: Aryl hydrocarbon
AUC: Area under the curve
CDC: Centers for Disease Control and Prevention
CI: Confidence interval
HR: Hazard ratio
IARC: International Agency for Research on Cancer
IOM: Institute of Medicine
NIOSH: National Institute for Occupational Safety and Health
NR: Not reported
OR: Odds ratio
PCP: Pentachlorophenol
PMR: Proportionate mortality ratio
PPB: Parts per billion
PPT: Parts per trillion
PSA: Prostate-specific antigen
RR: Relative risk
SD: Standard deviation
SIR: Standardized incidence ratio
SMR: Standardized mortality ratio
TCDD: 2,3,7,8-Tetrachlorodibenzo-*p*-dioxin
TCP: Trichlorophenol
TEQ: Toxic equivalency quotient
VA: Veterans Administration

## Footnotes

1 Throughout this paper, relative risk point estimates are followed by 95 % confidence intervals unless otherwise stated. Where confidence intervals were not reported by the authors, Fisher’s exact 95 % confidence intervals were calculated.
