# Supplementary material for: A critical review of the epidemiology of Agent Orange/TCDD and prostate cancer
Source: Eur J Epidemiol. 2014 Jul 27;29(10):667–723. doi: 10.1007/s10654-014-9931-2 (PMC4197347; doi:10.1007/s10654-014-9931-2)
Supplement: Supplementary file 1 — Supplementary material 1 (DOCX 176 kb) [file 10654_2014_9931_MOESM1_ESM.docx]

**Figure S1. Forest plot of prostate cancer relative risks in epidemiologic studies of Agent Orange/TCDD exposure**. Whiskers show 95% confidence intervals, except where not reported (indicated by *). HR: hazard ratio; MRR: mortality relative risk; OR: odds ratio; ppt: parts per trillion; RR: relative risk; SEA: Southeast Asia; SIR: standardized incidence ratio; SMR: standardized mortality ratio; TCDD: 2,3,7,8-tetrachlorodibenzo-*p*-dioxin; US: United States; VN: Southeast Asia military service in Vietnam.

**Figure S2. Forest plot of prostate cancer relative risks in epidemiologic studies of military service in Vietnam**. Whiskers show 95% confidence intervals, except where not reported (indicated by *). ADVA: Australian Department of Veterans’ Affairs; AIHW: Australian Institute of Health and Welfare; MOR: mortality odds ratio; MRR: mortality relative risk; OR: odds ratio; PC: prostate cancer; PR: prevalence ratio; RR: relative risk; SIR: standardized incidence ratio; SMOR: standardized morbidity odds ratio; SMR: standardized mortality ratio; SMRR: ratio of standardized mortality ratios; US: United States.

**Figure S3. Forest plot of prostate cancer relative risks in epidemiologic studies of occupational or accidental industrial exposure to TCDD**. Whiskers show 95% confidence intervals, except where not reported (indicated by *). 2,4-D: 2,4-dichlorophenoxyacetic acid; 2,4,5-T: 2,4,5-trichlorophenoxyacetic acid; HR: hazard ratio; MRR: mortality relative risk; ppb: parts per billion; RR: relative risk; SIR: standardized incidence ratio; SMR: standardized mortality ratio; TCDD: 2,3,7,8-tetrachlorodibenzo-*p*-dioxin; TEQ: toxic equivalency quotient for dioxins; US: United States.

**
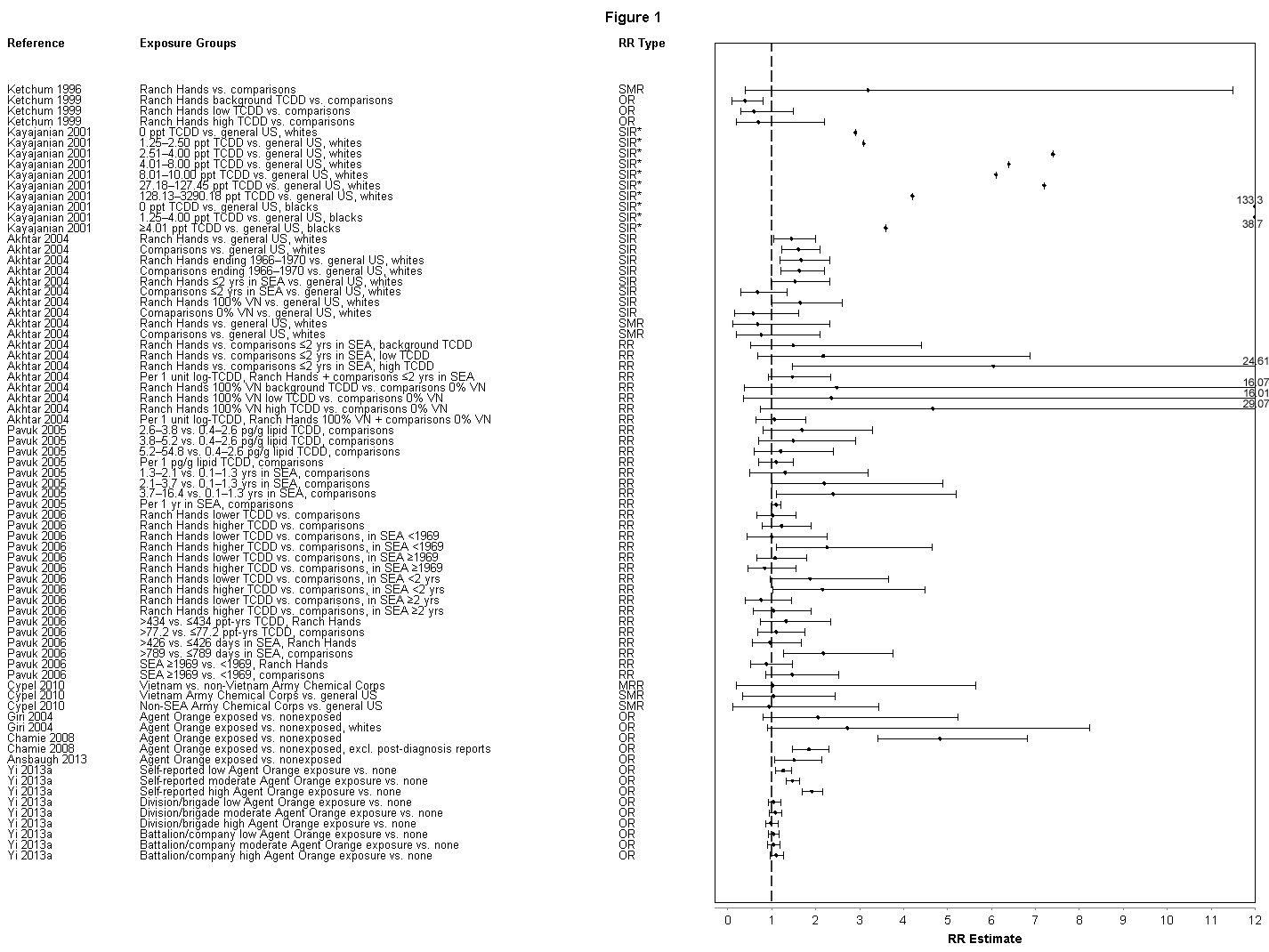
**


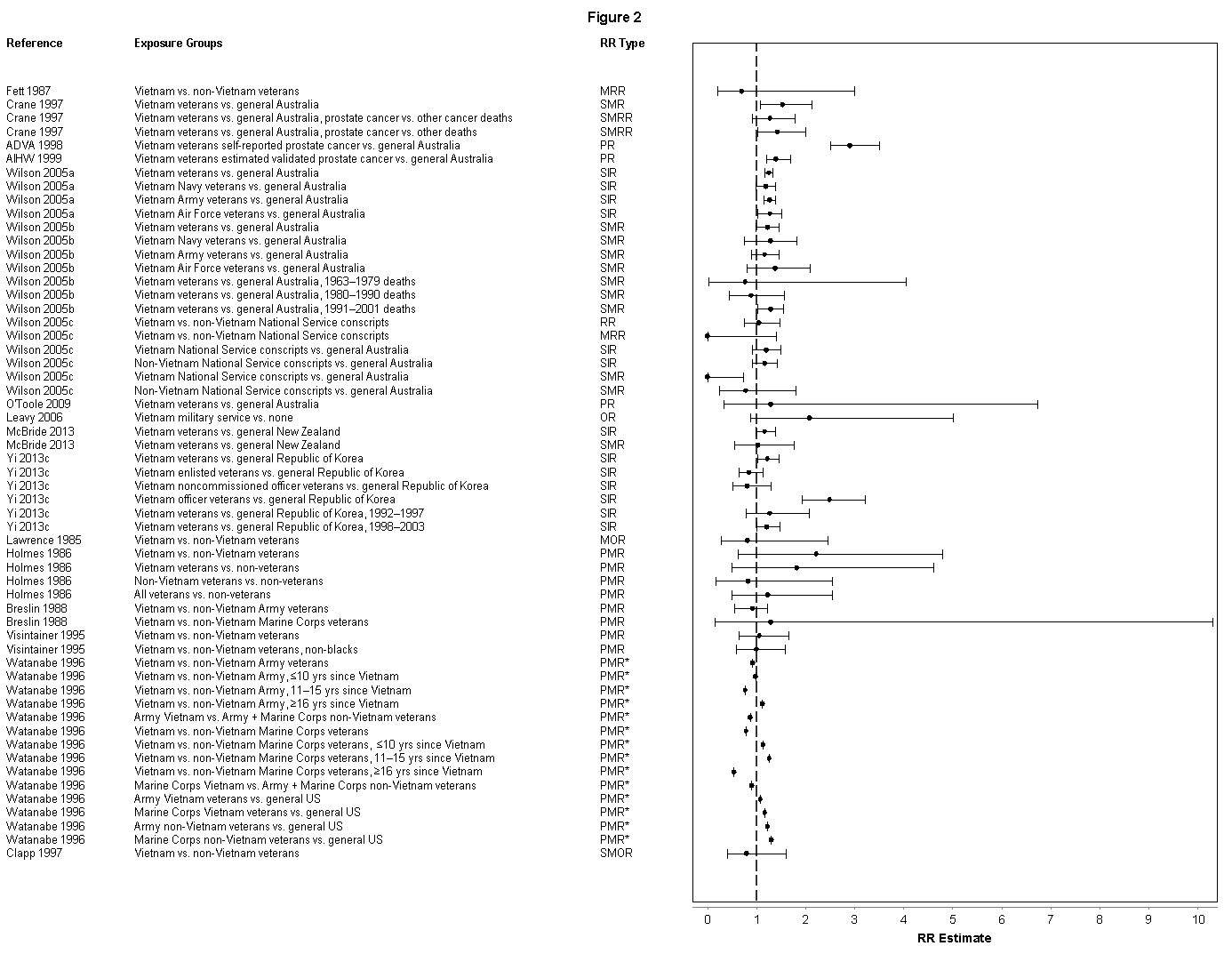


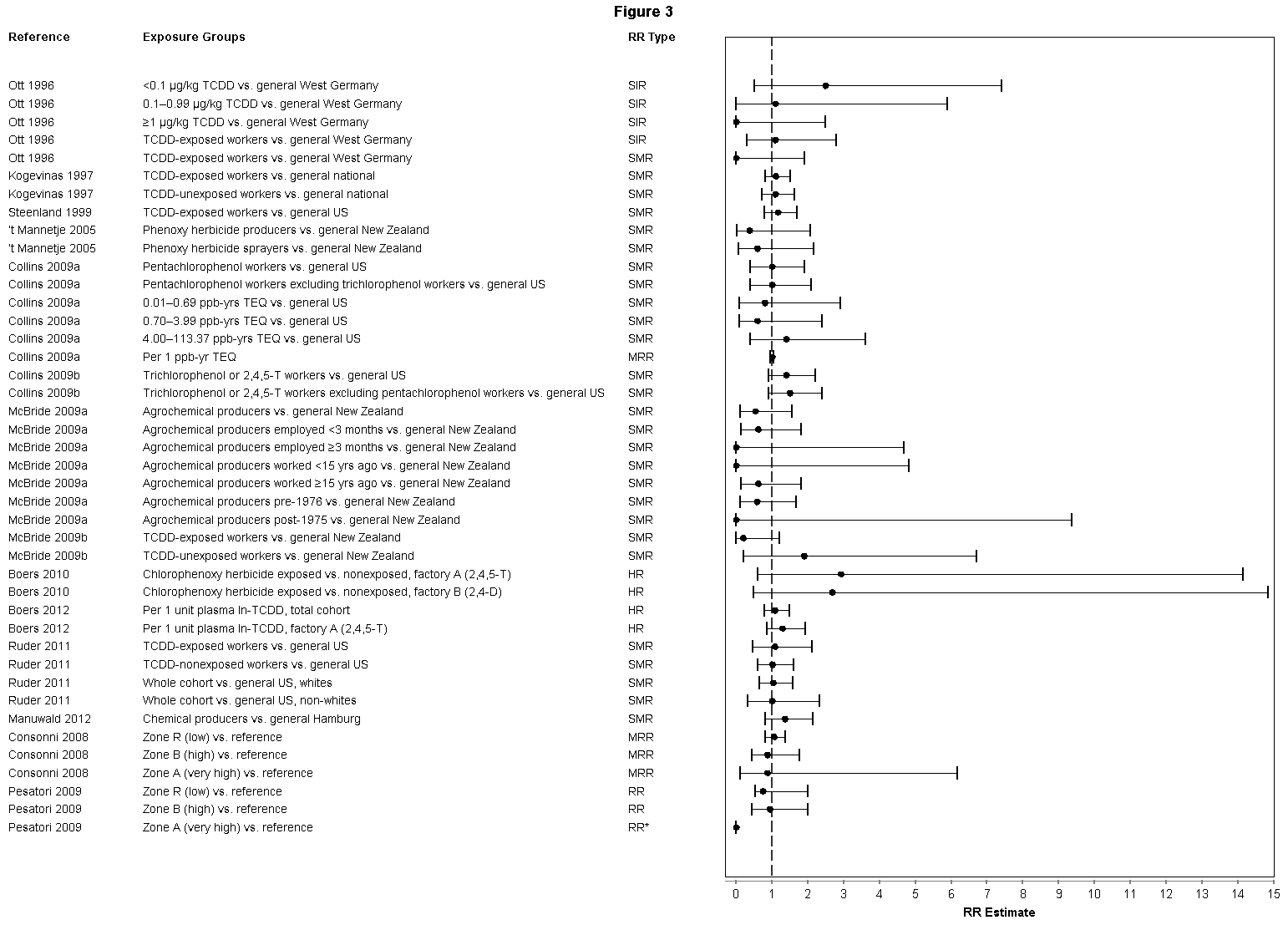


| **Table S1. Cohorts of herbicide production workers and sprayers included in the International Association for Research on Cancer/National Institute of Occupational Safety and Health (IARC/NIOSH) pooled analysis (Kogevinas et al., 1997)** | | | | | | |
| --- | --- | --- | --- | --- | --- | --- |
|  |  |  |  |  |  |  |
| **Country** | **Type** | **Sex** | **N TCDD-exposed** | **N TCDD-unexposed** | **Period of follow-up** | **Comments** |
| Australia | S | M | 1,840 | 0 | 1951–1983 |  |
| Austria | P | MF | 159 | 0 | 1971–1991 |  |
| Canada | S | M | 1,142 | 81 | 1950–1992 |  |
| Denmark | P | MF | 0 | 1,920 | 1947–1992 |  |
| Denmark | P | MF | 0 | 198 | 1951–1992 |  |
| Finland | P | MF | 62 | 0 | 1939–1991 |  |
| Germany | P | MF | 576 | 59 | 1956–1989 |  |
| Germany | P | MF | 1,307 | 42 | 1952–1989 | Updated (men only) by Manuwald et al., 2012 |
| Germany | P | MF | 313 | 179 | 1965–1989 |  |
| Germany | P | MF | 126 | 15 | 1951–1989 |  |
| Italy | P | MF | 205 | 0 | 1970–1991 |  |
| Italy | P | MF | 0 | 60 | 1967–1991 |  |
| The Netherlands | P | MF | 524 | 38 | 1955–1991 | Updated by Boers et al., 2010, 2012 (Factory A) |
| The Netherlands | P | MF | 0 | 419 | 1965–1991 | Updated by Boers et al., 2010, 2012 (Factory B) |
| New Zealand | P | MF | 782 | 0 | 1969–1990 | Updated by 't Mannetje et al., 2005; extended by McBride et al., 2009a, 2009b |
| New Zealand | S | MF | 699 | 0 | 1972–1990 | Updated by 't Mannetje et al., 2005 |
| Sweden | P | MF | 244 | 0 | 1965–1990 |  |
| United Kingdom | P | M | 0 | 1,565 | 1947–1990 |  |
| United Kingdom | P | M | 145 | 0 | 1960–1989 |  |
| United Kingdom | P | M | 572 | 369 | 1975–1991 |  |
| United Kingdom | P | M | 0 | 345 | 1963–1991 |  |
| United Kingdom | P | M | 0 | 271 | 1969–1991 |  |
| United Kingdom | P | M | * | * | 1969–1991 |  |
| United Kingdom | S | M | 0 | 1,992 | 1647–1990 |  |
| United States | P | M | 437 | 0 | 1951–1987 | Updated by Steenland et al., 2009 |
| United States | P | M | 96 | 0 | 1968–1987 | Updated by Steenland et al., 2009 |
| United States | P | M | 691 | 0 | 1961–1987 | Updated by Steenland et al., 2009 |
| United States | P | M | 354 | 0 | 1957–1987 | Updated by Steenland et al., 2009 |
| United States | P | M | 113 | 0 | 1961–1987 | Updated by Steenland et al., 2009 |
| United States | P | M | 121 | 0 | 1957–1987 | Updated by Steenland et al., 2009 |
| United States | P | M | 96 | 0 | 1960–1987 | Updated by Steenland et al., 2009 |
| United States | P | M | 452 | 0 | 1948–1987 | Updated by Steenland et al., 2009 |
| United States | P | M | 2,089 | 0 | 1942–1987 | Updated by Steenland et al., 2009; also studied by Collins et al., 2009a, 2009b and Ruder & Yin, 2011 |
| United States | P | M | 265 | 0 | 1949–1987 | Updated by Steenland et al., 2009 |
| United States | P | M | 163 | 0 | 1957–1987 | Updated by Steenland et al., 2009 |
| United States | P | M | 258 | 0 | 1953–1987 | Updated by Steenland et al., 2009 |
| Total |  |  | 13,831 | 7,553 |  |  |
| M: men; MW: men and women; P: herbicide production workers; S: sprayers; TCDD: 2,3,7,8-tetrachlorodibenzo-*p*-dioxin | | | | | | |
| * 479 workers could not be classified with regard to TCDD exposure | | | | | | |
